# Cryo-EM structures of ClC-2 chloride channel reveal the blocking mechanism of its specific inhibitor AK-42

**DOI:** 10.1038/s41467-023-39218-6

**Published:** 2023-06-09

**Authors:** Tao Ma, Lei Wang, Anping Chai, Chao Liu, Wenqiang Cui, Shuguang Yuan, Shannon Wing Ngor Au, Liang Sun, Xiaokang Zhang, Zhenzhen Zhang, Jianping Lu, Yuanzhu Gao, Peiyi Wang, Zhifang Li, Yujie Liang, Horst Vogel, Yu Tian Wang, Daping Wang, Kaige Yan, Huawei Zhang

**Affiliations:** 1 Shenzhen Institute of Advanced Technology, Chinese Academy of Sciences, 518055 Shenzhen, China; 2 Department of Biomedical Engineering, Southern University of Science and Technology, 518055 Shenzhen, China; 3 School of Life Sciences, Southern University of Science and Technology, 518055 Shenzhen, China; 4 Shenzhen Key Laboratory of Translational Research for Brain Diseases, The Brain Cognition and Brain Disease Institute, Shenzhen Institute of Advanced Technology, Chinese Academy of Sciences, Shenzhen, China; 5 Shenzhen-Hong Kong Institute of Brain Science-Shenzhen Fundamental Research Institutions, 518055 Shenzhen, Guangdong China; 6 University of Chinese Academy of Sciences, Beijing, China; 7 School of Life Sciences, The Chinese University of Hong Kong, Shatin, Hong Kong China; 8 Shenzhen Shuli Tech Co., Ltd, 518126 Shenzhen, Guangdong China; 9 Interdisciplinary Center for Brain Information, The Brain Cognition and Brain Disease Institute, Shenzhen Institute of Advanced Technology, Chinese Academy of Sciences, 518055 Shenzhen, Guangdong China; 10 Faculty of Life and Health Sciences, Shenzhen Institute of Advanced Technology, Chinese Academy of Sciences, 518055 Shenzhen, Guangdong China; 11 Department of Child and Adolescent Psychiatry, Shenzhen Kangning Hospital, Shenzhen Mental Health Center, Shenzhen, 518020 China; 12 Cryo-EM Facility Center, Southern University of Science and Technology, 518055 Shenzhen, Guangdong China; 13 Institut des Sciences et Ingénierie Chimiques (ISIC), Ecole Polytechnique Fédérale de Lausanne (EPFL), Lausanne, Switzerland; 14 Department of Orthopedics, Shenzhen Intelligent Orthopaedics and Biomedical Innovation Platform, Guangdong Provincial Research Center for Artificial Intelligence and Digital Orthopedic Technology, Shenzhen Second People’s Hospital, The First Affiliated Hospital of Shenzhen University, 518000 Shenzhen, China

**Keywords:** Cryoelectron microscopy, Molecular biophysics, Chloride channels, Electron microscopy

## Abstract

ClC-2 transports chloride ions across plasma membranes and plays critical roles in cellular homeostasis. Its dysfunction is involved in diseases including leukodystrophy and primary aldosteronism. AK-42 was recently reported as a specific inhibitor of ClC-2. However, experimental structures are still missing to decipher its inhibition mechanism. Here, we present cryo-EM structures of apo ClC-2 and its complex with AK-42, both at 3.5 Å resolution. Residues S162, E205 and Y553 are involved in chloride binding and contribute to the ion selectivity. The side-chain of the gating glutamate E205 occupies the putative central chloride-binding site, indicating that our structure represents a closed state. Structural analysis, molecular dynamics and electrophysiological recordings identify key residues to interact with AK-42. Several AK-42 interacting residues are present in ClC-2 but not in other ClCs, providing a possible explanation for AK-42 specificity. Taken together, our results experimentally reveal the potential inhibition mechanism of ClC-2 inhibitor AK-42.

## Abstract (web summary)

AK-42 was reported as a specific inhibitor of ClC-2 but its working mechanism is not clear. Here authors report cryo-EM structures of apo ClC-2 and in complex with AK-42, revealing how it inhibits ClC-2 specifically.

## Introduction

ClC proteins mediate the transfer of chloride ions across biological membranes and exert diverse physiological functions in organisms ranging from bacteria to humans^1^. ClC-2, one of the mammalian ClC proteins, is widely expressed in most tissues and plays key roles in many physiological processes, such as ion and water homeostasis, fluid transport and membrane excitability^2,3^. Dysfunction of ClC-2 in *Homo sapiens*, in the form of loss-of-function or gain-of-function mutations, has been shown to be closely related to a broad spectrum of diseases^4–9^. Loss-of-function ClC-2 mutations are closely related to diseases like leukodystrophy^1,7,10^. For example, A500V was one of the first identified mutations in leukodystrophy patients^8,11^. This mutation reduces the membrane localization of ClC-2 and impairs its stability and gating properties^8,11^. Co-expression with GlialCAM, an auxiliary subunit of ClC-2 mainly found in glia, can partially rescue ClC-2 A500V channel activity by targeting ClC-2 to the plasma membrane and by altering its gating properties as well^1,7,8,12,13^. Recent studies have shown that the full development of leukodystrophy requires the disruption of ClC-2 in both astrocytes and oligodendrocytes, whereas the development of retinal or testicular degeneration requires disruption of ClC-2 only in retinal pigment epithelial cells or Sertoli cells, revealing the cell-specific manner of ClC-2-related pathologies^7^. In contrast, primary aldosteronism was reported to be associated with gain-of-function mutations in ClC-2^4,5,14^. Studies show that the R180Q mutation of mouse ClC-2 (corresponding to R172Q in human ClC-2) can increase chloride efflux, elevate aldosterone levels and blood pressure, and eventually cause primary aldosteronism in mice^14^. In line with this report, the mutation R172Q in human ClC-2 was observed in patients with early-onset primary aldosteronism^5,15^. Functional analysis showed that transfection of R172Q mutant in HAC15 cells results in constitutive depolarization of the cell compared with that of wild-type ClC-2, which increases the open probability of ClC-2 channels and further enhances chloride efflux at the resting membrane potential^5,15^. This predisposes zona glomerulosa cells to a depolarization state and eventually increases aldosterone production^5,15^. Other mutations in human ClC-2, such as M22K, G24D, Y26N, K362del, and S865R, have also been reported to be related to familial hyperaldosteronism type II^4,5^. These mutations disrupt the channel activities of ClC-2 and cause abnormal Cl^−^ currents in a similar manner to that of the R172Q mutation^15^. However, due to the lack of experimental structural information, how these mutations affect the normal function of ClC-2 has not been elucidated at the atomic level.

According to their localization and working mechanism, members of the mammalian ClC family can be further classified into two subgroups: the Cl^−^ channels (ClC-1, ClC-2, ClC-Ka and ClC-Kb) and the Cl^−^/H^+^ transporters (ClC-3, ClC-4, ClC-5, ClC-6, and ClC-7)^1^. Cl^−^ channels reside in the plasma membrane and conduct Cl^−^ passively, whereas Cl^−^/H^+^ transporters mainly localize in the endo/lysosomal membrane and transfer Cl^−^ via proton-coupled secondary active transport^1^. Despite their differences, mammalian ClC proteins share a common architecture^16–18^. Structural alignments of ClC proteins also show close similarity across different species^16,18^. To date, ClC proteins with solved structures are present in dimer form in a roughly C2 symmetrical manner with common architectural features^16–21^. Each protomer consists of an N-terminal domain, a transmembrane helix domain (TMD) and two C-terminal cystathionine-β-synthase (CBS) domains, and has an independent Cl^−^ conduction channel pathway with three chloride-binding sites named S_ext_, S_cen,_ and S_int_, respectively^1,16,18,21^. The opening and closing of ClC transporters are mainly regulated by protonation states of the gating glutamate Glu_gate_ such as E232 in human ClC-1^16,18,21–23^. When protonated, the side chain of Glu_gate_ swings out from the S_cen_ site and thus opens the channel. In contrast, it swings into the S_ext_ or S_cen_ site when deprotonated and thus closes the channel^16,19,21,24,25^. ClC channels have been considered as broken transporters^18,24,26,27^. The degraded transporter model proposes that ClC channels and transporters are evolutionarily conserved with some subtle differences, such as different conformations of the gating glutamate, wider pore diameter in the intracellular side of the channel, and reduced chloride affinity in the binding sites^16,18^.

ClC-2 functions as an inward-rectifying chloride channel and its activity is regulated by many physiological factors, such as hyperpolarized voltage, osmotic cell swelling, extracellular protons, intracellular Cl^−^ and ATP^1,27–35^. The gating of ClC-2 is controlled by both protopore gates (fast gating) and a common gate (slow gating)^36–38^. The common gate is shared by two protomers and mainly regulated by the cytoplasmic region containing the N-terminal domain and C-terminal CBS domains. Deletion or point mutations of the CBS2 domain mainly affect the common gate and eventually result in faster activation and deactivation of ClC-2^36,37,39^. Similarly, deletion of the N-terminal domain also causes faster kinetics of the ClC-2 channel^40^. Accordingly, mutations around these regions cause abnormal ClC-2 activity and are closely related to human diseases such as primary aldosteronism^4,5,15^. In contrast, the protopore gates are located in each protomer. Similar to other ClC proteins, three residues were identified in ClC-2 in the potential chloride binding sites along the channel (S162, E205 and Y553 in human ClC-2; S168, E211 and Y559 in rat ClC-2; S170, E213 and Y561 in mouse ClC-2)^18,41,42^. Structural modeling and electrophysiological recording experiments demonstrated that E213 and Y561 in mouse ClC-2 are energetically coupled in the closed state^41^. Upon occupied by Cl^−^ ion, the Y561-E213 coupling is disrupted by electrostatic and steric repulsion, E213 is then repelled outward to adopt an outward-facing conformation^41^. This observation supports the idea that the pore occupancy of permeant anions regulates the activation of ClC-2^43,44^. Molecular dynamics simulation of rat ClC-2 fast gating demonstrated that the rotation of the S168 region plays a critical role in initializing the opening of the ClC-2 channel, and the gating glutamate also adopts an outward conformation when the channel is open^42^. However, the conformations adopted by human ClC-2 Glu_gate_ during the transport cycle have not been experimentally elucidated.

Due to the importance of ClCs, many studies have been performed to design, screen, and optimize the modulators of ClCs to regulate their activity^45–49^. However, ClC-2 is almost insensitive to classical ClC inhibitors such as 9-anthracenecarboxylic acid (9-AC) and 4,4’-Diisothiocyanostilbene-2,2’-disulfonic acid (DIDS)^1,2,50,51^. A peptide toxin, GaTx2, was isolated from scorpion venom and found to inhibit ClC-2 channels at a dissociation constant of 20 pM^45,46^. However, GaTx2 has no effect on open ClC-2 channel^45,46^. As a derivative of meclofenamate, AK-42 was recently reported to be a potent and specific inhibitor of the ClC-2 chloride channel^47^. AK-42 inhibits ClC-2 with great selectivity (over 1000-fold compared to its close homolog ClC-1) and high efficacy, with an IC_50_ of 17 nM^47^. Computational docking results have identified several residues in the specific interaction between ClC-2 and AK-42^47^. Although some mutagenesis studies have been performed, the experimental structural basis for the blocking mechanism still needs further investigation.

Here, we determined the structures of apo ClC-2 and its complex with AK-42 using cryogenic electron microscopy (cryo-EM). The Cl^−^ channels in each promoter were analyzed, and key residues were identified. Interface analysis between ClC-2 and AK-42 also revealed a hydrophobic binding pocket critical for their interaction. Structure-guided molecular dynamics and electrophysiological recordings further confirm those identified binding residues. Several AK-42 interacting residues in the pocket are present only in ClC-2 but not in other ClCs, possibly explaining the specificity of AK-42 to ClC-2. Our study provides insight into the details of ClC-2 chloride channel blockade by the specific inhibitor AK-42.

## Results

### Overall architecture of ClC-2 and its complex with the specific inhibitor AK-42

**Fig. 1:**
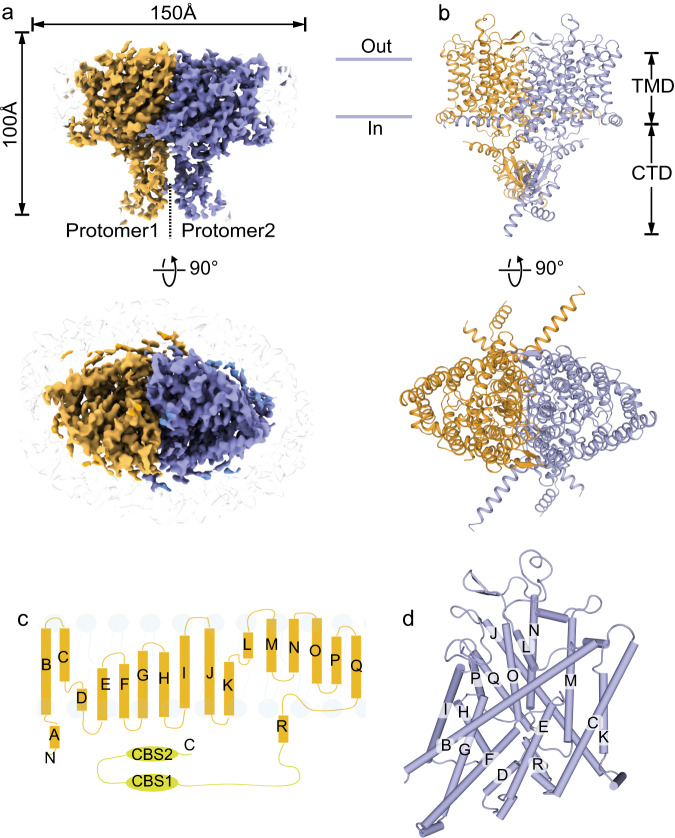
Cryo-EM structure of a full-length human ClC-2 homodimer. **a** Front view and top view of ClC-2 cryo-EM density map in homodimer form. Two protomers are shown in brown and purple, respectively. **b** Front view and top view of the ClC-2 atomic model. Color scheme is the same as in (**a**). The obtained ClC-2 structure can be divided into transmembrane domain (TMD) and the cytosolic C-terminal domain (CTD). **c** Overall topology of human ClC-2 with transmembrane helices (labeled as A-R) and two cytosolic cystathionine beta synthase (CBS) domains. **d** Three-dimensional arrangement of transmembrane helices of one protomer as shown in (**c**).

The full-length human ClC-2 protein was expressed in HEK293F cells, solubilized by the mild detergent N-Dodecyl-β-D-maltoside (DDM), and purified via affinity chromatography and size exclusion chromatography (Supplementary Fig. 1). Cryo-EM data were collected using a 300 kV Titan Krios microscope equipped with a K2 camera and processed according to the procedure described in Methods section and in Supplementary Fig. 2. Cryo-EM structures of the apo ClC-2 and its complex with highly specific inhibitor AK-42 were both determined at 3.5 Å resolution (Fig. 1a, Supplementary Figs. 3, 4a–c, Supplementary Table 1). These cryo-EM structures represented a homodimer of ClC-2 proteins. The overall architectures of these two structures were generally the same, except for the additional cryo-EM density of the AK-42 inhibitor in the complex (Supplementary Figs. 3, 4d). The two maps displayed clear densities of residue side chains in the transmembrane domain (TMD) (Supplementary Fig. 4c, d). However, the cytosolic C-terminal domain (CTD) had lower resolution, suggesting that the two tandem-linked CBS domains are quite flexible. Both TMD and CTD domains appeared to participate in the dimerization of ClC-2 proteins. The refined atomic model contains residue numbers from 89 to 560, fitting well within the density of our TMD map, but lacking the flexible N- and C-terminal regions (Fig. 1b). Similar to other ClC family proteins, the TMD domain of each protomer in the ClC-2 dimer had a triangular prism-like architecture composed of 18 α-helices (named Helix A-R) (Fig. 1c, d), and each subunit had its own chloride permeation pathway across the membrane. Structural alignment of ClC-2 TMD domain with that from ClC-1 or bClC-K resulted in root mean square deviation (RMSD) values of 0.845 Å and 1.007 Å for all atoms, respectively.

### Chloride transport pathway and gating mechanism of ClC-2

**Fig. 2:**
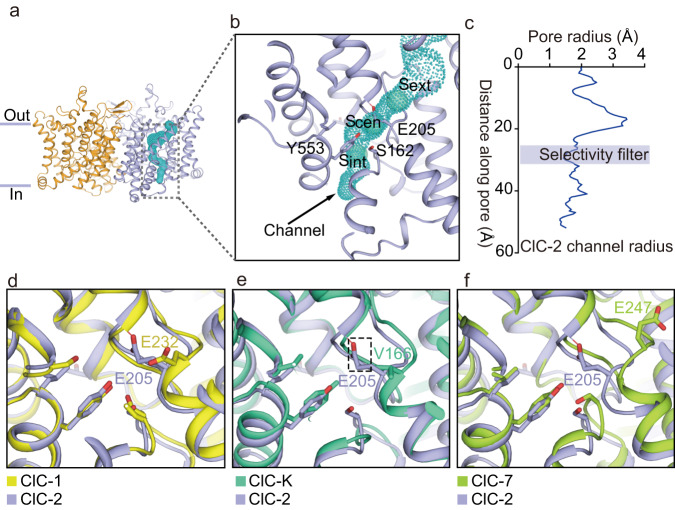
Transmembrane domain of the ClC-2 channels in the apo state. **a** Overall structure of the transmembrane domain of ClC-2 homodimer. Protomers are colored in brown and purple, respectively. The chloride channel was identified by CAVER plugin^52^ in PyMOL (https://pymol.org/2/) and shown as cyan dots. Only one chloride channel was shown for simplicity. **b** Enlarged map of the chloride channel, where cyan balls represent three conserved putative chloride ion binding sites inferred from sequences (from top to bottom: S_ext_, S_cen,_ and S_int_). The regions of the selectivity filter (Y553 and S162) and the fast gate (E205) are labeled. **c** The pore radius along the chloride pathway from the extracellular side to the cytosol is visualized in the chart. The selectivity filter formed by S162, Y553 and E205 is highlighted. E205 was mutated to alanine during the pore radius calculation to reserve the pore connectivity. Source data are provided as a Source Data file. **d–f** Comparison of the channel gating of human ClC-2 with (**d**) human ClC-1 (PDB ID 6COY)^18^, (**e**) bovine ClC-K (PDB ID 5TQQ)^16^, and (**f**) human ClC-7 (PDB ID 7JM7)^21^. ClC-2 was colored in purple. Human ClC-1, bovine ClC-K and human ClC-7 were colored in yellow, green and light green, respectively.

To unravel the transport pathway of ClC-2, the anion-permeable channel in one ClC-2 protomer was identified and visualized using PyMOL (https://pymol.org/2/) via the CAVER plugin^52^ (Fig. 2a, b). Three putative chloride binding sites (S_ext_, S_cen,_ and S_int_)^16^ are mapped in the ClC-2 structure and shown as spheres inside this channel (Fig. 2b). The chloride binding sites were similar to those of previously reported from *Escherichia coli* ClC-Ec and human ClC-1^1,18,23^. Studies on human ClC-1 identified several conserved residues that control the ion selectivity and permeability of its substrates, including the pore filter residues S189 and Y578, and the gating glutamate E232^18^. In ClC-2, the corresponding residues are S162, Y533 and the gating glutamate E205, respectively. These three residues form a narrow constriction in the ion permeation pathway with a radius of 1.4–1.5 Å, which is comparable to that of human ClC-1^18^ (E205 was mutated to alanine computationally to reserve the pore connectivity during the pore radius calculation with reference to the previous report^18^), indicating that those three residues might also play critical roles in the gating of ClC-2 (Fig. 2c). Previous studies showed that ClC-2 was permeable to anions but not cations^1,2,43,53^ with a preference for Cl^−^ over other halides (Br^−^, I^−^), which is typical among ClC transporters and channels^2,53,54^. ClC-2 is also permeable to organic anions such as SCN^−^^43^. These substrate preferences may be determined by the narrow channel and a set of filter residues (S162, Y533 and E205) (Fig. 2c).

Gating glutamate residue such as E232 in human ClC-1 plays a critical role in the regulation of ClC proteins^18^. Here, in the middle of the human ClC-2 channel, the side chain of the gating glutamate E205 occupies the central chloride binding site (S_cen_), precluding the binding of chloride, indicating that our structure most likely represents the closed state of the human ClC-2 channel. We further compared the gating glutamate E205 from human ClC-2 with those from other ClC proteins such as human ClC-1, bovine ClC-K and human ClC-7. The side chain of E232 from human ClC-1 is far away from the chloride transport pathway, representing the open state of the human ClC-1 fast gate^18^ (Fig. 2d). However, there is no gating glutamate in bovine ClC-K^16^. The equivalent residue V166 in bovine ClC-K has a small side chain that does not block the channel (Fig. 2e), indicating that bovine ClC-K adopts an open state^16^. For human ClC-7 transporter, the side chain of gating glutamate E247 points to the extracellular vestibule and does not occupy the S_cent_ site, although human ClC-7 adopts an occluded state due to other narrow constrictions along the channel^21^ (Fig. 2f).

### Blocking of ClC-2 by the specific inhibitor AK-42

ClC-2 is a widely distributed Cl^−^ channel in humans, and its dysfunction is linked with severe human diseases^4–7^. Therefore, the development of specific inhibitors to modulate its function is medically important. Despite the high similarity of ClC channels, specific inhibitors such as 9-AC for ClC-1 and the *N*-arylated benzimidazole derivative BIM1 for ClC-Ka have been reported^50,55^. A high-affinity peptide GaTx2 from scorpion venom was reported to block ClC-2 with a dissociation constant of approximately 20 pM^45,46^, but it does not block ClC-2 channels in the open state^45,46^. Recently, AK-42 was reported to function specifically against human ClC-2^47^. Although the original study provided several computational docking results using AK-42 with a predicted rat ClC-2 model, experimental structures are still missing^47^. Here, we report the cryo-EM structure of human ClC-2 in complex with the AK-42 compound, which revealed similar but not identical AK-42 binding patterns from the docking results in the previous report^47^.

**Fig. 3:**
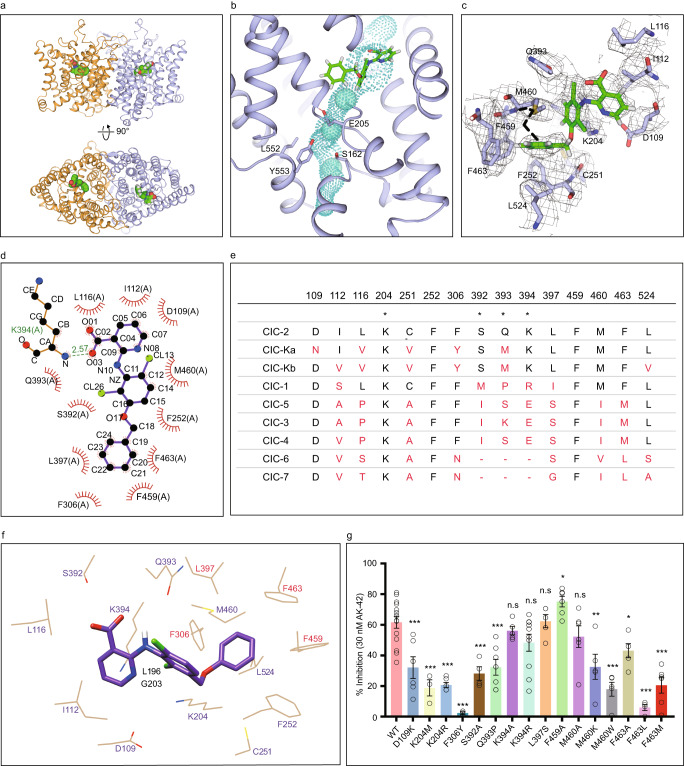
Transmembrane domain of the AK-42 inhibitor-bound ClC-2. **a** Front view and top view of the AK-42 bound ClC-2 transmembrane domain. Two protomers are shown in brown and purple, respectively. AK-42 is shown in green sphere. **b** Close-up view of the chloride channel with AK-42 bound. The AK-42 inhibitor is located above the S_ext_ site and blocks the Cl^−^ channel. Residues S162, E205, L552, Y553 are shown in stick. AK-42 are shown in green stick. **c** Conformational change of M460 induced by AK-42. The black dashed line indicates the side chain of M460 in the apo state, which moves upward upon AK-42 bound. Densities of surrounding residues are shown in gray. **d** Interactions between ClC-2 and AK-42 were analyzed by the LigPlot^+^ program^56^. K394 and AK-42 form a hydrogen bond with a distance of 2.57 Å. **e** ClC-2 residues around the AK-42 inhibitor and homologous residues in other human ClC proteins were extracted and compared. * indicates the three most critical residues in the binding with AK-42 identified in per-residue energy decomposition shown in Supplementary Fig. 7i. **f** Mapping of identified key residues to the cryo-EM model. Residues identified both in the previous docked models and in the cryo-EM model are labeled in purple color. Residues identified only from the docked models are labeled in black color. Residues identified only from the cryo-EM model are shown in red color. Numbering of residues is according to human ClC-2. **g** Measurement of the AK-42 inhibition effect on wild-type ClC-2 and its mutants. ***P < 0.001; **P < 0.01; *P < 0.05; ns, not significant. Data are presented as the mean ± SEM. Exact *n* values and *P* values are listed in Supplementary Table 3. *N* values represent biologically independent cells recorded. Normality was assessed by the D’Agostino-Pearson test. Statistical analyses of percent inhibition between WT and mutants were performed by two-tailed Student’s *t* test. Source data are provided as a Source Data file.

To further investigate the binding mode of AK-42, we synthesized AK-42 and determined the complex structure of ClC-2/AK-42 using cryo-EM (Fig. 3a, Supplementary Figs. 2b, 3a–d, 4d, 5). AK-42 bound with ClC-2 from the extracellular side and occupied the outer vestibule of the chloride pathway. AK-42 was located just above the conserved external chloride binding site S_ext_ and directly blocked the permeation pathway of anions (Fig. 3b). Structural alignment using apo ClC-2 and its complex with AK-42 showed that their overall structures were nearly identical, with a RMSD of 0.619 Å for all atoms. However, the side chain of M460 showed an obvious shift to induce the phenyl ring of AK-42 to fit into a hydrophobic pocket (Fig. 3c). The corresponding residue M485 in human ClC-1 likely forms the constriction of ClC-1 and affects its throughput for chloride ions (Supplementary Fig. 6)^18^. LigPlot^+^ program^56^ was further used to analyze the binding interface between ClC-2 and AK-42, and identify key residues for their interactions (Fig. 3d). The aforementioned hydrophobic pocket was formed by M460, F252, F459, F463, F306, and L397. Moreover, the AK-42 heterocycle also had hydrophobic interactions with I112 and L116. The carboxyl group of AK-42 appeared to form a hydrogen bond with the K394 main chain amino group (Fig. 3d). Additional potential hydrogen bonds and π-cation interactions between AK-42, K204 and S392 can be identified by PLIP software^57^ (Supplementary Fig. 3d).

To better understand the molecular basis of AK-42 selectivity on human ClC-2, we aligned human ClC protein sequences and other ClC members via ESPript3^58^ (Supplementary Fig. 6). The ClC-2 residues involved in AK-42 binding were selected and listed in Fig. 3e, together with their corresponding residues from other ClC proteins. Among these 15 selected residues, ClC-2 differs by at least 5 residues (highlighted in red in Fig. 3e) from other ClC proteins. Further, K204, S392, Q393 and K394 were ranked in top residues that contribute more binding energy for the interaction between ClC-2 and AK-42, as demonstrated below in the “*Molecular dynamics simulations and MM/PBSA free energy calculations*” section. These sequence variances intuitively showed the structural basis of AK-42’s specificity to ClC-2.

### Molecular dynamics simulations and MM/PBSA free energy calculations

To further characterize the interaction between ClC-2 and AK-42, molecular dynamics simulations and MM/PBSA free energy calculations^59^ were performed for our AK-42-bound ClC-2 structure. Energy minimization, as well as canonical ensemble *(NVT)* and isothermal-isobaric ensemble *(NPT)* equilibration, were first performed to set up this system. The potential energy eventually stabilized at −1.41 × 10^6^ kJ mol^−1^ after 1200 steps. Temperature, pressure, and density were calculated to be 300 K, 0.9 bar, and 1008 kg m^−3^ (SPCE water density), respectively, indicating that this system was successfully set up (Supplementary Fig. 7a–d). The RMSD value between the equilibrated structure and cryo-EM structure leveled off at approximately 0.22 nm (2.2 Å), and the radius of gyration value remained almost unchanged, indicating that the overall structure was quite stable during simulation. The highest RMS fluctuation values were approximately 0.4 nm at atom 5188 (corresponding to residue A217), indicating flexibility in this region (Supplementary Fig. 7e–g).

To quantify the contributions of key residues to the interaction between ClC-2 and AK-42, MM/PBSA free energy calculations were performed. Usually, van der Waals, electrostatic and nonpolar interactions dominate the energy distribution. The binding free energy components are shown in Supplementary Fig. 7h. The electrostatic energy showed the most favorable contributions to the total binding free energy, followed by the van der Waals energy and the nonpolar solvation energy. The above findings revealed that the electrostatic energy was the most important contributor to protein-ligand binding, probably because of the electrostatic field stabilizing this complex. These results correspond to the general results found in the structural studies (Fig. 3). MM/PBSA decomposition analysis can usually provide in-depth insight into protein-ligand interactions. The last step was the MM/PBSA per-residue decomposition, which is demonstrated in Fig. 7i and reveals that K394, S392, Q393 and K204 play the most important roles in protein-ligand interactions due to their larger contributions relative to other residues.

### Electrophysiological validations of the key residues for ClC-2 and AK-42 interactions

Residues involved in the interactions between rat ClC-2 and AK-42 have been assigned in the previous computational study, collectively from all the docked AK-42 poses^47^. Many of those identified residues are consistent with that from our experimental model, including D109, I112, L116, K204, C251, F252, S392, Q393, K394, M460 and L524 (residue numbering is according to human ClC-2) (Fig. 3d, f, Supplementary Fig. 4d)^47^. Additionally, some residues such as G203 are identified previously but not in our model, whereas residues such as F306 and F463 are identified in our model but not in the previous models^47^. Compared with the docked models, phenyl ring of AK-42 in our cryo-EM model adopts a similar but not identical conformation due to a rotation relative to other parts of AK-42. That might be the possible reason that residues such as F306 and F463 are only identified in our model (Fig. 3f, Supplementary Fig. 8a–f)^47^. It is worth noting that for each specific docked model, there will be less identified residues (those confirmed by electrophysiological recordings as discussed below), compared with that from our cryo-EM model (Supplementary Fig. 8g). Other than the differences of techniques used (computational docking method using predicted models versus cryo-EM), those discrepancies might also come from subtle differences between different species (rat ClC-2 versus human ClC-2). To further characterize and confirm the ClC-2 residues interacting with AK-42 (Fig. 3d), different mutants of human ClC-2 were constructed at the specific residues identified either in our present experimental model or the previous computational one, except residues such as F252 due to the extreme low expression level of their mutants. Wild-type human ClC-2 and its mutants were expressed in CHO-K1 cells, followed by channel activity recordings using the whole-cell patch clamp technique in the presence or absence of AK-42 according to the procedures described previously^47^. The inhibition kinetics with a time course for wild-type ClC-2 and all mutants are shown in Supplementary Fig. 9 and Supplementary Table 2. The current traces before and after AK-42 application are shown in Supplementary Fig. 10. The percent inhibition (as defined in the Methods section) was shown in Fig. 3g and Supplementary Table 3, allowing us to evaluate the contribution of individual ClC-2 residues to AK-42 binding. A lower percent inhibition value implies less stable binding between the mutated residue and AK-42, which suggests a more significant role of the corresponding residue in the interactions between ClC-2 and the inhibitor.

As depicted in Fig. 3g, application of 30 nM AK-42 induces approximately 60 percent inhibition of wild-type human ClC-2, which is comparable with the results of the previous study using rat ClC-2^47^. We then compared the electrophysiological results of the site-specific mutations Q393P, K204M, K204R, K394R and K394A in human ClC-2 with previous results obtained from computer modeling of rat ClC-2 (corresponding residues are K400R, Q399P, K210M and K210R in rat ClC-2)^47^. The mutations Q393P, K204M and K204R in human ClC-2 show similar effects of reducing the percent inhibition, compared with the corresponding ones in rat ClC-2 reported previously^47^. K394R and K394A showed a slightly lower but not significant percent inhibition of AK-42 than wild-type ClC-2, comparable to that in the previous study^47^. Considering that the O = C group of AK-42 forms hydrogen bond with the N-H group in the main chain of K394 rather than the side chain (Fig. 3d), it is likely that mutations of K394 result in only subtle differences, as the N atom remains unchanged.

Furthermore, some contact residues have been identified in our present model but not in the previous study^47^, including F306 and F463, which form hydrophobic contacts with the phenyl ring of AK-42 (Fig. 3d). As shown in Fig. 3g, the percent inhibition values of the F306Y, F463A, F463M and F463L mutations were significantly reduced compared with that of wild-type ClC-2, implying critical roles of this hydrophobic pocket in stabilizing the AK-42 inhibitor. Roles of F306 in AK-42 binding can be confirmed by F306Y, although the current of F306N mutant behaved distinctly to that of wild-type ClC-2 (Supplementary Fig. 11a). Interestingly, the mutation F459A in the hydrophobic pocket shows a higher percent inhibition value than wild-type ClC-2, possibly due to the steric hindrance effect of the bulky side chain of phenylalanine, indicating potential directions for the further optimization of ClC-2 inhibitors based on the structure of AK-42. The G203 residue (corresponding to G209 of rat ClC-2 in the previous report^47^) was identified in the previous report but not in our model, as it is quite far from AK-42 in our structure, although electrophysiological validation was not reported previously (Supplementary Fig. 8g). In our study, the “Initial” current of G203A mutant without AK-42 application seems to bear no resemblance to wild-type ClC-2 currents (Supplementary Fig. 11a), and its roles in AK-42 binding may still need further confirmation. Considering the pivotal role of K204, it is also possible that G203 may indirectly affect AK-42 binding via the adjacent K204. Other residues, such as D109, S392, M460 and L524 (corresponding to D115, S398, M466 and L530 from rat ClC-2 in the previous report^47^), were identified in both our results and the previous report^47^ but were not verified by electrophysiological experiments previously. Thus, we tested the related mutations D109K, S392A, M460A, M460K and M460W in patch clamp experiments. The results shown in Fig. 3g indicate that most of them showed a lower percent inhibition value than the wild-type ClC-2, suggesting their potential roles in mediating the interaction between ClC-2 and AK-42. Among them, the side chain of M460 was shifted dramatically upon binding AK-42 (Fig. 3c). Whereas for L524S, its current pattern is distinct from that of wild-type ClC-2, and whether it plays a role in AK-42 binding still needs further exploration (Supplementary Fig. 11a).

The current traces of some mutants such as G203A, F306N and L524S showed distinct features from those of wild-type ClC-2 (Supplementary Fig. 11a). The typical current magnitude of wild-type ClC-2 and most mutations was continually increased during the 250 ms time course with higher hyperpolarization voltage applied, whereas the current profiles of G203A, F306N and L524S were somewhat flattened or even slightly increased (for L524S) during the time course. The whole-cell current-voltage (I-V) curves were also significantly different from that of wild-type ClC-2 (Supplementary Fig. 11b). To test whether those currents are unspecific leak or endogenous currents, iodide block experiments and control experiments using un-transfected cells and empty-vector transfected cells are performed. The currents for those controls are negligible compared with cells transfected with ClC-2 as shown in Supplementary Fig. 11b. For the iodide block experiments, 80 mM cesium iodide (CsI) was firstly used for iodide block experiments accordingly to previous studies^2^. The current of cells transfected with wild-type ClC-2 was increased transiently and then decreased after application of CsI (Supplementary Fig. 11c). The transiently increasing of currents may be because of the replacement of CsCl by CsI in the extracellular solution. To test this, we then used 20 mM CsI for iodide block experiments. Although the amplitude of transient increasing was reduced compared with that of 80 mM CsI application, the inhibition effect of CsI seems not obvious. Current amplitudes of G203A, F306N and L524S mutants with application of 80 mM and 20 mM CsI are also not inhibited significantly by CsI (Supplementary Fig. 11c). Taken together, currently we cannot rule out the possibility that those currents of G203A, F306N and L524S are likely from unspecific leak or endogenous currents (Supplementary Fig. 11a–c), future work will be needed to clarify the nature of current contributions for those mutations. After careful inspection of those residues in the apo ClC-2 structure, we found that the G203, F306 and L524 residues are located close to the ClC-2 channel near the selectivity filter and this might explain why those mutants dramatically change the current profile of ClC-2 (Supplementary Fig. 11d).

To summarize, many of the identified residues for the interaction between ClC-2 and AK-42 are consistent with the previous report^47^, whereas residues such as F306 and F463 were identified from our model. Our data will provide valuable validation and improvements to the previous model^47^ and guide the further development of specific inhibitors against ClC-2 based on the structure of AK-42.

## Discussion

ClC transporters utilize the gating glutamate to control their opening and closing, which are intimately associated with Cl^−^ ion conduction^16,21–23^. Based on previous studies, the negative charges on the carboxyl group of the gating glutamate and chloride ions compete with each other to fulfill the electrostatic balance in the proposed transport cycle^24,26,27,60^. Although considered as broken transporters, ClC channels also have a gating glutamate (E232 in human ClC-1 and E205 in human ClC-2), except for ClC-K where glutamate is replaced by valine^16,18,24,26,27^. Previous studies have shown that the gating glutamate together with a tyrosine within the selectivity filter are energetically coupled in the closed state of ClC-2^41^. The binding of Cl^−^ disrupts the coupling and results in the outward-facing conformation of the gating glutamate when the ClC-2 channel is open^41,42^. In our study, the position of the Glu_gate_ side chain in ClC-2 occupies the S_cen_ site in the selectivity filter and exhibits significant differences from the glutamate side chains of ClC-1^18^ (Fig. 2). The closed conformation of ClC-2 and the open conformation of ClC-1 may reflect the fact that the Glu_gate_ of these ClC channels adopts at least two different states during the Cl^−^ transport cycle^18,41^, and our structure of apo ClC-2 has experimentally captured one of the important states, facilitating understanding of the working mechanism of the ClC channel. Previous functional studies indicate that ClC-1 and ClC-2 exhibit distinct voltage-dependent properties. ClC-1 was activated by membrane depolarization, whereas ClC-2 was closed at depolarized potentials and activated by membrane hyperpolarization^61,62^. Those results are consistent with the structural observations that ClC-1 was open whereas ClC-2 was closed at 0 mV where their structures were determined^18^.

In the paper proposing the computational docking model of AK-42, experimental structural basis for ClC-2/AK-42 interactions and explanations for activity variances of AK-42 scaffold derivatives were not available^47^. Here, based on our structures, interaction details between ClC-2 and AK-42 were experimentally confirmed, which may explain the half-maximal inhibitory concentration IC_50_ data from previous reports^47^ (Fig. 3d, Supplementary Figs. 4d, 12). The AK-24 molecule is very similar to AK-42 except for the nitrogen atom on the heterocycle of AK-42, which forms potential hydrogen bonds with the K204 residue (Supplementary Fig. 3d). Due to the difference, AK-24 was found to be 5 times less potent than AK-42. In line with this observation, the K204R mutation significantly reduced the percent inhibition value of AK-42 (Fig. 3g). Other molecules have modifications on the phenyl ring of AK-42, which influence their interactions with the hydrophobic pocket formed by M460, F252, F459, F463, F306, and L397. The related mutations M460A, M460K, M460W, F306Y, F463A, F463M and F463L thus affected the binding of AK-42 to ClC-2 (Fig. 3g). Moreover, molecules with hydrophilic modifications (AK-37, 38, 39, 40, 41, 43, and 44) showed much less activity than hydrophobic-modified inhibitors (AK-45, 46, 47, and 48), further supporting the importance of the identified hydrophobic pocket (Fig. 3d). In the previous docked models, the phenyl ring of AK-42 adopts a different conformation and cannot explain the mutation data for F306 and F463 (Fig. 3g)^47^. Other derivatives, such as AK-49, with multiple substitutions of the phenyl ring of AK-42, also have severely decreased interactions with the hydrophobic pocket. The inhibitor AK-50 has a longer linker than AK-24 and AK-42, which may destabilize the intermolecular coordination. Thus, the lower inhibition effects of AK-49 and AK-50 may be due to steric hindrance (Supplementary Fig. 12).

**Fig. 4:**
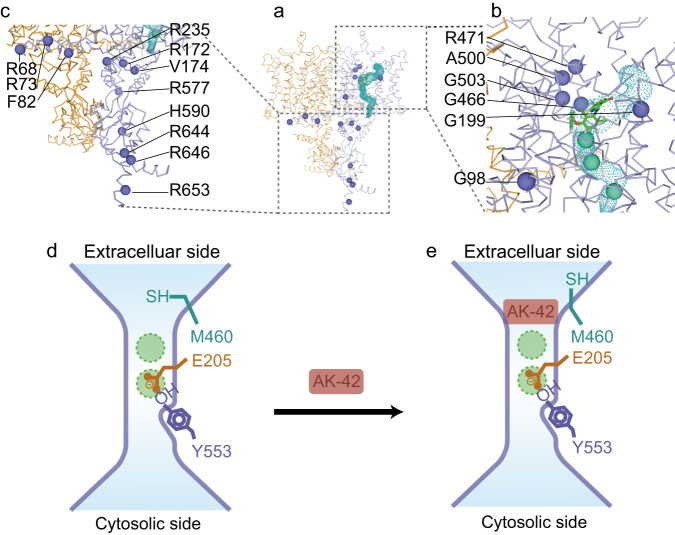
Mapping of disease-causing mutations in the ClC-2 structure. **a** Labeling of genetic mutation sites (purple color balls) on one protomer of the ClC-2 dimer. The chloride channel and AK-42 inhibitor were also shown. **b** Close-up view of disease-related mutation sites in the transmembrane domain of ClC-2. Those sites include G98, G199, G466, R471, A500 and G503. **c** Close-up view of disease-related mutation sites in the para-membrane regions and CBS domains. Those sites include R68, R73, F82, R172, V174, R235, R577, H590, R644, R646 and R653. **d**, **e** Proposed model to explain the direct blocking mechanism of AK-42 in ClC-2. Upon binding of AK-42 to ClC-2, the side chain of M460 residue adopts another conformation, however the overall structure of ClC-2 does not change significantly. Residues E205, Y553, M460 and two putative chloride binding sites are shown.

To date, over 50 disease-causing genetic mutations have been reported for ClC-2, including missense, nonsense, deletion, and insertion mutations^4–9^. These mutations can result in loss or gain of function of ClC-2 channels. Impaired ClC-2 may lead to leukodystrophy or atrial fibrillation^7,8^, whereas gain-of-function mutations are closely related to primary aldosteronism^4,5,14^. All the mutations above may directly influence the chloride permeation pathway, indirectly change the gating of this channel, or cause abnormal expression levels of ClC-2 on the plasma membrane. To highlight this, we selected representative missense mutations visible in our full-length cryo-EM map and labeled them as spheres in one protomer of the ClC-2 model (Fig. 4a–c). These mutations can be classified into three categories including mutations in the protomer-protomer interface area, mutations in the ion channel area and mutations in the cytoplasmic area. Among them, G98R^9^ is located near the interface area and may influence the protomer-protomer interaction. G199A^63^, R172Q^5,15,64^, V174S^11^, G466E^9^, R471C^9^, A500V^8,11^, and G503R^65^ are located around the ion channel pathway. They can be further classified into two groups depending on their localizations. The G199A, G466E, R471C, A500V, and G503R sites are near the extracellular vestibule of the chloride channel, while R172Q and V174S are located near the intracellular vestibule. Here, amino acid substitution might directly influence the anion translocation pathway. For example, G199 is located in the loop between α helices E and F, and may be involved in swelling-accelerated voltage gating^63^. The inward current of the G199A mutation failed to increase during hypotonic swelling^63^. For R172Q, current-clamp recording experiments demonstrated that this mutation causes gain of function of ClC-2 and depolarization of the plasma membrane, eventually increases aldosterone production, and is closely related to early-onset primary aldosteronism^5,15,64^. In contrast, the mutation A500V significantly reduces the ClC-2 level at the plasma membrane and affects its channel gating, thus resulting in loss of function of ClC-2 and eventually causing leukoencephalopathy^8,11^. Some deletion or indel mutations in this region, such as M200fs (fs indicates frameshift)^34,66,67^ and K362del^5^, cause a premature form of ClC-2 or in-frame deletion of residue K362 and ultimately disrupt the normal function of ClC-2. Other mutations are located in the cytoplasmic area, including R68H^63^, R73H^9,68^, R235Q^69^, R577Q^33,69,70^, H590P^71^, R644C^69^, R646Q^63^, and R653T^33,70^. This area contains an N-terminal inactivation domain that can regulate the gating of ClC-2^15,40,72^ and two CBS domains^15,16^ that can bind with ATP^33,34^ and is important for voltage-dependent channel activation^39^. In our study, ATP was not added in the sample solution used for cryo-EM, thus our ClC-2 structure may represent the state in the absence of ATP. These mutations in this area might affect ATP binding or the voltage-dependent ClC-2 activation process and eventually affect ClC-2 channel properties. For example, the R68H mutation showed significantly faster activation kinetics than wild-type ClC-2^63^, whereas the R235Q and R577Q mutations near the CBS domains showed accelerated deactivation kinetics of the ClC-2 channel compared with wild-type ClC-2^69^. Some other mutations in this region such as M22K^5^, G24D^4,15^, Y26N^5^, R725W^63^, R747H^63^, G715E^33,34,66,70^, R753T^73^, and S865R^5^, also affect ClC-2 channel regulation, although they are not directly observed in our structures.

ClC-2 is only weakly sensitive to classical Cl^−^ channel blockers such as 9-AC^1,50^. AK-42 is a high affinity and specific small-molecule inhibitor targeting ClC-2, but its binding mode with ClC-2 still needs experimental validation^47^. Based on our findings, we propose a direct blocking model to explain how AK-42 inhibits the channel activity of ClC-2 (Fig. 4d, e). After binding the ligand AK-42, the overall structure of the ClC-2 channels does not change significantly, except for the side chain of methionine at position 460. The movement of M460 induces the fitting of a phenyl ring into the hydrophobic pocket formed by M460, F252, F459, F463, F306, and L397. Other residues such as K204, S392 and K394 are also involved in the interaction between ClC-2 and AK-42 through potential hydrogen bonds and π-cation interactions (Supplementary Fig. 3d). Many of these residues are unique and explain AK-42’s specificity against ClC-2 (Fig. 3d–f). In general, AK-42 may inhibit ClC-2 channel by directly blocking its ion conduction pathway (Fig. 4d, e).

In conclusion, we have presented the structures of human ClC-2 in its apo form and in complex with the specific inhibitor AK-42. Our study provides a working model of ClC-2 that describes the blocking mechanism of AK-42 and lays the foundations for the development of interventions against ClC-2 related diseases.

## Methods

### Protein expression and purification

A codon-optimized complementary DNA clone for full-length *ClC-2* (Gene ID: 1181) from *Homo sapiens* was synthesized and cloned into a pCAG-MCS-TEV-EGFP-2S-V93A vector using *XhoI* and *XbaI* restriction sites. The recombinant vector contained ClC-2, tobacco etch virus (TEV) protease cleavage site, EGFP and Strep-tag II from 5’ to 3’. Mutants of ClC-2 were constructed using the Q5 Site-Directed Mutagenesis Kit (New England Biolabs, E0554S). Primers are listed in Supplementary Table 4. All clones were confirmed by sequencing. This plasmid was mixed with PEI MAX 40 K (Polysciences, 24765-1) at a 1:3 ratio for 30 min and then used to transfect HEK293F cells at a cell density of (2–3) × 10^6^ ml^−1^. HEK293F suspension cells were cultured in SMM 293T-II medium (Sino Biological Inc., M293TII-1) in the presence of 8% CO_2_ and 60% humidity. After 72 h of incubation at 37 °C, the HEK293F cells from 2.3 L of culture were harvested by centrifugation at 6370 × *g* for 10 min.

The collected cells were resuspended in 100 ml of lysis buffer (20 mM HEPES and 150 mM NaCl, pH 7.4) containing 0.2 mM PMSF, and this resuspension was subjected to 4 cycles of high-pressure homogenization at a pressure of 1080 MPa. Subsequently, large particles of cell debris were removed from the cell lysate by low-speed centrifugation at 6370 × *g* for 10 min. The membrane fractions in the supernatant were extracted by ultracentrifugation at 58, 540 × *g* for 2 h. Then, the membranes containing the target protein were solubilized in 40 ml of lysis buffer supplemented with 1% N-Dodecyl-β-D-maltoside and 0.1% cholesteryl hemisuccinate (Anatrace, D310-CH210) for 12 h at 4 °C. The solubilized membrane proteins were diluted 5 times with lysis buffer and further separated by centrifugation at 15,000 × *g* for 20 min. After centrifugation, the supernatant was bound to 8 ml of Strep-Tactin resin (IBA Lifesciences, 2-1201-010) for 2 h, which had been equilibrated with a buffer containing 20 mM HEPES pH 7.4, 150 mM NaCl, 0.2% DDM, and 0.02% CHS (buffer A). Strep affinity chromatography was performed by washing this resin with 40 ml of wash buffer containing 20 mM HEPES pH 7.4, 150 mM NaCl, 0.2% DDM, 0.02% CHS, 10 mM ATP, and 10 mM Mg^2+^. The bound ClC-2 proteins were released from these beads by overnight TEV protease digestion (1:20 molar ratio) and eluted with buffer A. To remove the TEV protease, protein samples were further purified with high-affinity Ni-NTA resin. The retrieved protein was concentrated to 0.5-0.6 ml using Amicon Ultra centrifugation units with a 30 kDa cutoff (Millipore) and then applied to a Superose 6 300/10 GL column (GE Healthcare) that had been equilibrated with 20 mM HEPES pH 7.4, 150 mM NaCl, and 0.02% GDN. The peak fractions were finally pooled, concentrated to ~3.3 mg ml^−1^, and immediately used for cryo-EM grid preparation.

### Chemical synthesis of AK-42

The ClC-2 inhibitor AK-42, also known as 2-((3-(benzyloxy)−2,6-dichlorophenyl)amino)nicotinic acid, was synthesized from SM1 through 4 steps as shown below in the schematic diagram. During AK-42 synthesis, mass spectra were obtained on a Shimadzu LC-2020 spectrometer in direct-inlet mode. High-performance liquid chromatography was performed on an Agilent-1200. 1H-NMR spectra were recorded on a Varian UNITY 400 (400 MHz) in dimethyl sulfoxide (DMSO)-d6 with tetramethylsilane as an internal standard. Details of synthesis can be found in Supplementary Fig. 13.

### Cryo-EM sample preparation

Peak fractions of ClC-2 from a Superose 6 column were concentrated to ~3.3 mg ml^−1^ for apo samples and 4.5 mg ml^−1^ for AK-42 samples. For AK-42 samples, the inhibitor was added to ClC-2 at a final molar ratio of AK-42:ClC-2 = 4:1 with 0.5% DMSO due to the poor solubility of AK-42. The mixture was incubated at 18 °C for 8 h. Then, 3 µl of each sample was applied to glow-discharged holey carbon grids (Quantifoil R1.2/1.3) and blotted for 2 s, followed by plunging into precooled liquid ethane using an FEI Vitrobot Mark IV (Thermo Scientific).

### Cryo-EM image acquisition and data processing

Sample grids were loaded onto a 300 kV Titan Krios G3i electron microscope (Thermo Fisher) equipped with a Gatan K2 Summit detector (Gatan). Movie stacks were recorded using SerialEM software^74^ at a magnification of 165k in super-resolution mode, with a total electron dose of 50 e Å^−2^ for 32 frames and a defocus range from −1.0 to −2.5 μm. After real-time motion correction and dose weighting by MotionCor2^75^, the calibrated pixel size was 0.829 Å. In total, 7374 and 8951 movies were collected for the apo and AK-42 datasets, respectively.

The dose-weighted and motion-corrected micrographs were then imported into RELION-3.1^76^ to determine their defocus values and CTF parameters via Gctf v.1.18^77^. Then, 1,612,725 and 1,741,519 particles were automatically picked from the apo and AK-42 datasets, respectively. The extracted particles were binned 4 times and transferred to several rounds of two-dimensional classification, and then 659,250 (apo) and 483,328 (AK-42) selected particles were re-extracted without binning. The selected particles were used to generate a 3D initial model in cryoSPARC-3.1^78^. These models and particles were then input into Relion-3.1 for global and local 3D classifications. A total of 43,510 (apo) and 44,153 (AK-42) selected particles were next subjected to 3D refinement. These refinement steps produced 4.3 Å (apo) and 4.7 Å (AK-42) maps. Then, soft masks of the transmembrane domain (TMD) were used for local refinement steps, and the final resolutions of these TMD maps were 3.5 Å for both maps, according to the gold-standard Fourier shell correlation (FSC) curve with a threshold of 0.143.

### Atomic model building

The initial monomer model of ClC-2 was generated using AlphaFold2^79^, where its flexible loop regions (CBS domain) were deleted before fitting into the cryo-EM density map of ClC-2. As the density of the CBS domain was not good enough for model building, we only manually adjusted the transmembrane domain of the model in Coot^80^ and performed model refinement in PHENIX^81^. Detection of the channel was performed using the Caver plugin^52^ of PyMOL (https://pymol.org/2/), while E205 was mutated to Ala to prevent blocking of the Cl^−^ pathway by the side chain of Glu_gate_. Figures were prepared in PyMOL and UCSF Chimera^82^. To prevent potential overfitting, we used a similar method for model validation to that in the previous report^83^. The FSC curves between the PDB models and the final full maps or the summed half-maps were named FSC_model_ and FSC_sum_, respectively. Then, the FSC_work_ between the random shaken (up to 0.5 Å) PDB models and 1st unfiltered half-maps were determined in Phenix refinement, and FSC_free_ represents the value between these refined shaken models and the 2nd unused unfiltered half-maps.

### Energy minimization

Energy minimization (EM) is a common technique to minimize the total energy of the receptor (protein) and ligand, which involves locating the energy minimum of all the atoms of the simulated system under specified conditions. Based on the initial unfavorable structure, the energy minimization program searches for the local minimum mathematically in 3D Cartesian coordinates using algorithms such as the steepest descent method, conjugate gradient method, Monte Carlo, hash table, bubble sort or L-BFGS. Then, we added solvent and charge and ensured the exclusion of steric hindrance. 15, 205 water molecules are added to the system. The water molecules totally cover the protein-ligand complex. The net charge on the protein was neutralized by Cl^−^ ions. 128 Dipalmitoylphosphatidylcholine lipid molecules were added around ClC-2 proteins. The final systems contained 61, 584 atoms. The box size used was 80 × 80 × 110 Å^3^. The system reaches equilibrium after 3000 steps.

### Equilibration procedure

NVT and NPT equilibration was performed before molecular dynamics simulation. NVT equilibration followed the NVT ensemble, which ensured that the particle number, system volume, and system temperature were constant. T then reached an equilibrated value. Normally 50–100 ps is used for the length of equilibration, in our case, 100 ps. NPT equilibration followed an NPT ensemble, where particle number, system pressure, and system temperature were constant. Pressure and density reached equilibrium during the simulation. V-rescale thermostat is employed for NVT equilibrium and Parrinello-Rahman barostat is employed for NPT equilibrium.

### Molecular dynamics simulation

We obtained the Cartesian coordinates from cryo-EM structures at 3.5 Å resolution. Membrane systems were built using the membrane building tool CHARMMGUI^84^. All the simulated systems were assigned for CHARMM36m force field^85^, and the ligand was assigned for CHARMM CGenFF force field^86^. After energy minimization and equilibration procedures, we carried out unbiased molecular dynamics simulation using Gromacs^87^. Periodic boundary condition was applied. All the production runs were carried out under the NPT ensemble. The production of simulations lasted for 5 ns and five replicates were used to ensure reproducibility. VMD was used to check the running trajectory^88^. Xmgrace software [https://plasma-gate.weizmann.ac.il/Grace/] was used to plot the data.

During a molecular dynamics (MD) simulation, RMSD and RMSF were used as two common indicators that measure the spatial changes of biomacromolecules. Since we care about the structure variation from the starting structure, we used the RMSD defined in Eq. (1) to describe the total discrepancy with respect to the reference conformation. A flattening of the RMSD curve indicates that a protein has equilibrated.

1 $${{{{{{\rm{RMSD}}}}}}}=\sqrt{\frac{{\sum }_{j=0}^{N}[{m}_{j}*{({X}_{j}-{Y}_{j})}^{2}]}{M}}$$

RMSF, as defined in Eq. (2), is a per-atom quantity and describes an atom’s fluctuation over the whole trajectory. It is used to evaluate each atom’s flexibility or vibration over an entire simulation course.

2 $${{{{{{\rm{RMSF}}}}}}}=\sqrt{\frac{1}{T}{\sum }_{t=1}^{T}{\sum }_{j=1}^{N}{\left({x}_{j}\left(t\right)-\bar{{x}_{j}}\right)}^{2}}$$

The moment of inertia of a body about an axis can sometimes be represented by the radius of gyration. We defined the radius of gyration here as the imaginary distance from the centroid where the cross-sectional area was focused at a point to obtain the same moment of inertia. It is formulated as in Eq. (3):

3 $$k=\sqrt{\frac{I}{m}}$$

### MM/PBSA per-residue energy decomposition

Since the molecular mechanics Poisson–Boltzmann surface area (MM/PBSA) method^59^ is accurate and less computationally costly than alchemical free energy methods, it has become a popular method for predicting binding free energy. The free energy of binding (Δ*G*_binding_) describes the degree to which a ligand transitioning from the solvated mode to the protein-bound mode is favored. It can be formulated as in Eq. (4):

4 $$\Delta {G}_{{{{{{{\rm{binding}}}}}}}}=\Delta H-T\Delta S$$

where Δ*G*_binding_ denotes the binding free energy, and Δ*H* denotes the enthalpy change. It can be decomposed into three terms: (1) the molecular mechanical energy (Δ*E*MM); (2) the Poisson−Boltzmann energy (Δ*G*_PB_); and (3) the nonpolar solvation energy (Δ*G*_nonpolar_). Hence, Δ*G*_binding_ can be rewritten as in Eq. (5):

5 $$\Delta {G}_{{{{{{{\rm{binding}}}}}}}}=\left(\Delta {E}_{{MM}}+\,\Delta {G}_{{PB}}+\,\Delta {G}_{{{{{{{\rm{non}}}}}}}{\mbox{-}}{{{{{{\rm{polar}}}}}}}}\right)-T\Delta S$$

The summation of the Poisson-Boltzmann energy and the nonpolar solvation energy is usually referred to as Δ*G*_PBSA_. Δ*E*_MM_ consists of the internal energy, van der Waals energy, and Coulombic energy calculated using the molecular mechanics force field. The aim of MM/PBSA computation is to identify the most important residues with the highest binding affinity to a target protein. This calculation is performed in explicit solvent MD simulations.

The computation of the nonpolar solvation free energy requires calculation of the solvent-accessible surface area (SASA). It has been found that the nonpolar solvation free energy has a linear relationship with SASA, as described in Eq. (6):

6 $$\Delta {G}_{{{{{{{\rm{non}}}}}}}{\mbox{-}}{{{{{{\rm{polar}}}}}}}}=\gamma {SASA}+\beta$$

### Electrophysiological recordings

Whole-cell recordings of CHO-K1 cells were performed 24 h after transfection at room temperature (22–25 °C) as described previously with minor modifications^47^. In brief, cells were recorded in a RC-26GLP recording chamber (Warner Instruments, USA) perfused with extracellular solution (ECS) composed of (in mM) 148 CsCl, 10 HEPES, 2 CaCl2, and 100 D-mannitol (pH 7.3-7.4 with CsOH). Borosilicate recording pipettes (3–6 MΩ) were filled with an internal solution composed of (in mM) 146 CsCl, 5 EGTA, 10 HEPES, 5 NaF, and 60 D-mannitol (pH 7.3-7.4 with CsOH). Solutions were applied and exchanged via gravity-based tubing (flow rate: 3 ml min^−1^) with manually operated valves. The initial current recording (“initial”) began 2 min after whole-cell mode was achieved and was elicited with a 0-mV, 20-ms holding potential followed by 250-ms voltage steps from −100 to +20 mV in 10-mV increments with a tail pulse to +80 mV for 100 ms. Each step was run every 5 s. After AK-42 treatment for 3 to 4 min (“30 nM AK-42”), the currents were monitored with the same protocol. Then, after 3 to 4 min of washout (“washout”) with ECS, the currents were recorded again. Currents were measured as the mean currents in the last 10 ms of the −100 mV voltage step relative to the baseline current of the 0-mV holding step. The current inhibition was calculated as follows: % Inhibition = (*I*_initial_-*I*_AK_)/I_initial_ × 100%, where *I*_initial_ and *I*_AK_ are the currents measured before and after treatment with 30 nM AK-42, respectively. For iodide block experiments, 80 mM or 20 mM CsCl in ECS was substituted with 80 mM or 20 mM CsI, respectively. We also tested the effect of vehicle (0.1% DMSO) on the current inhibition, and the results showed little effect (Supplementary table 5). Experiments in which the initial currents induced at −100 mV were larger than 100 pA and in which the inhibition calculated using initial currents differed from the inhibition calculated using washout currents by less than 30% were included for analyses. Signals were acquired with Multiclamp 700B and Digidata 1550B (Molecular Devices, USA). Data were filtered at 2 kHz and sampled at 10 kHz. All the data were recorded using Clampex 11.2 and analyzed with Clampfit 11.2 software (Molecular Devices, USA).

### Statistical analysis

Each data point in the patch clamp experiments was repeated at least three times. Data are presented as the mean ± SEM. Error bars represent the SEM. Statistical analyses were performed with Student’s *t* test (two-tailed) in GraphPad Prism 7.0 (GraphPad Software Inc.) to compare differences in mean values. Normality was assessed by the D’Agostino–Pearson test.

### Reporting summary

Further information on research design is available in the Nature Portfolio Reporting Summary linked to this article.

## Supplementary information

Supplementary Information

Reporting Summary

## Data Availability

The cryo-EM maps of human ClC-2 were deposited in the Electron Microscopy Data Bank under accession numbers EMD-33169 (Apo full length), EMD-33223 (Apo TMD domain) and EMD-34202 (AK-42 bound TMD domain). The corresponding atomic coordinates were deposited in the RCSB Protein Data Bank under accession numbers 7XF5, 7XJA and 8GQU, respectively. Atomic coordinates mentioned in this work can be accessed with PDB codes 6COY (human ClC-1), 5TQQ (bovine ClC-K) and 7JM7 (human ClC-7). The source data underlying Figs. 2c, 3g and Supplementary Figs. 1, 7, 11b are provided as a Source Data file. Source data are provided with this paper.

## Source data

Source Data
