# Supplementary material for: Cryo-EM structures of ClC-2 chloride channel reveal the blocking mechanism of its specific inhibitor AK-42
Source: Nat Commun. 2023 Jun 9;14:3424. doi: 10.1038/s41467-023-39218-6 (PMC10256776; doi:10.1038/s41467-023-39218-6)
Supplement: Supplementary file 1 — Supplementary Information [file 41467_2023_39218_MOESM1_ESM.pdf]

Supplementary Information for

Cryo-EM structures of ClC-2 chloride channel reveal the blocking mechanism of its specific inhibitor AK-42

Tao Ma *et al*

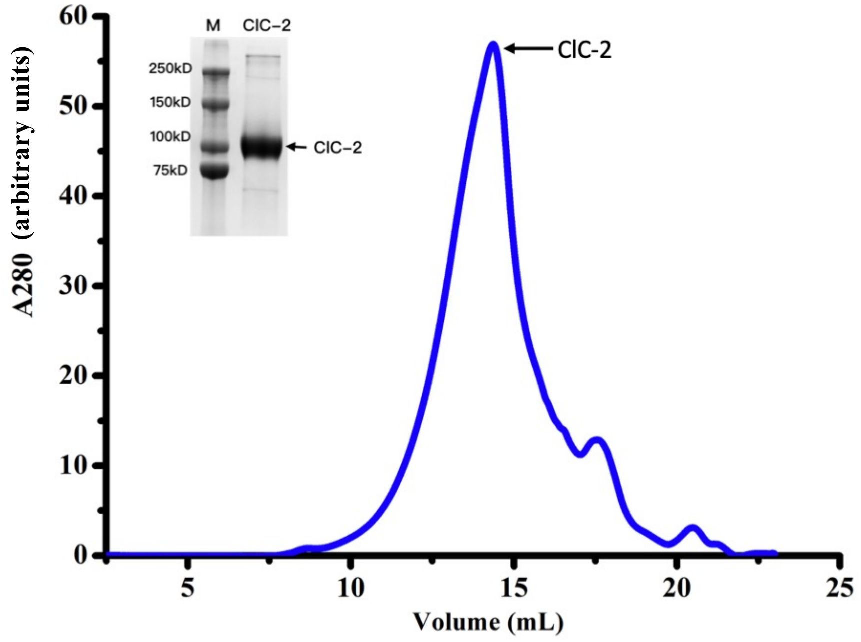

Supplementary Figure 1. Preparation of ClC-2 protein. ClC-2 was expressed using HEK293F cells and purified by affinity chromatography and size-exclusion chromatography. ClC-2 was eluted at about in 14.4 mL from Superose 6 column. Sodium dodecyl sulphate-polyacrylamide gel electrophoresis was used to confirm the presence and purity of ClC-2. M: Marker. The source data of size-exclusion chromatography profile and uncropped gel are provided as a Source Data file. The experiments have been repeated for three times with similar results.

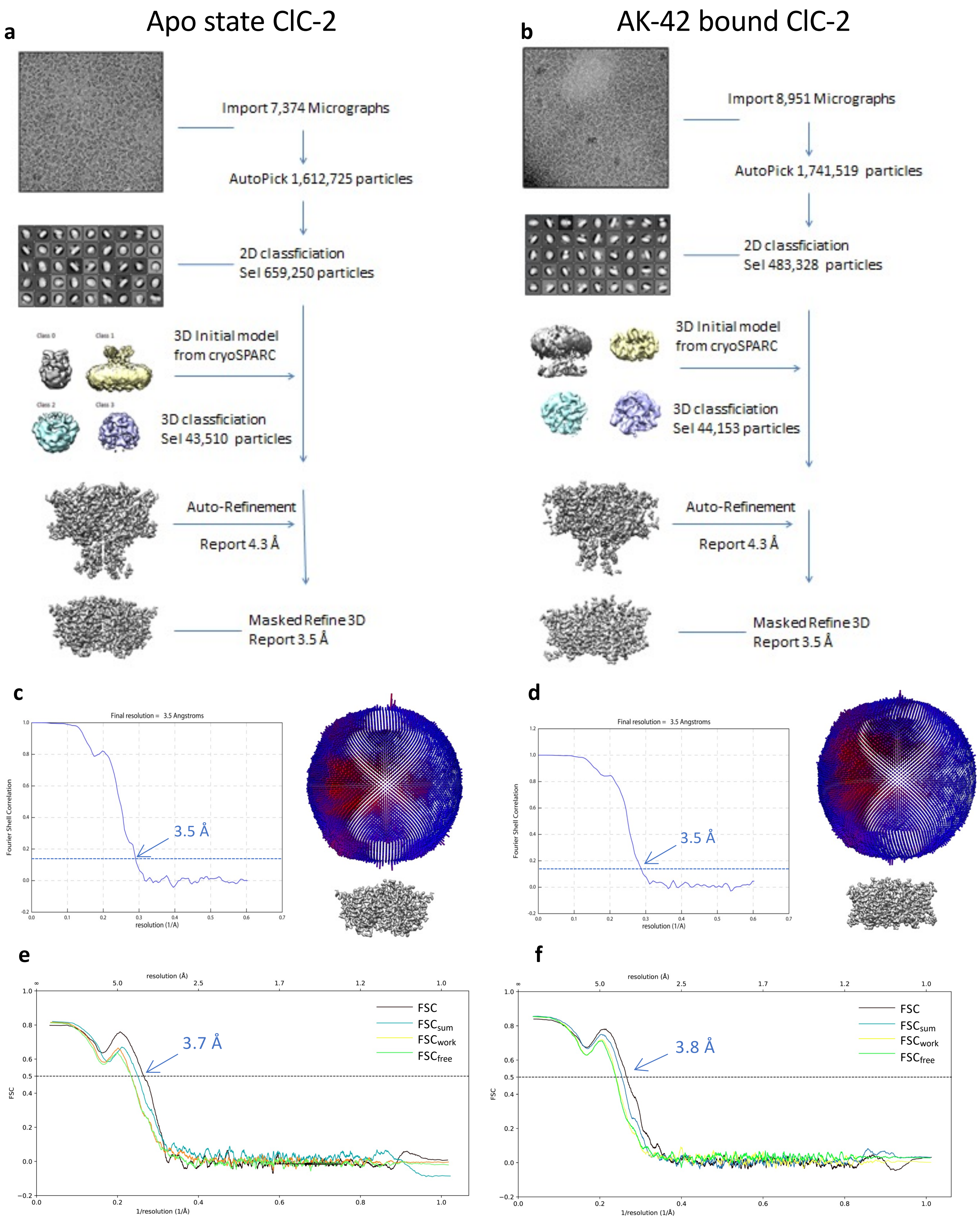

a
Apo state ClC-2
Import 7,374 Micrographs
AutoPick 1,612,725 particles
2D classficiation
Sel 659,250 particles
3D Initial model
from cryoSPARC
3D classficiation
Sel 43,510 particles
Auto-Refinement
Report 4.3 Å
Masked Refine 3D
Report 3.5 Å
b
AK-42 bound ClC-2
Import 8,951 Micrographs
AutoPick 1,741,519 particles
2D classficiation
Sel 483,328 particles
3D Initial model
from cryoSPARC
3D classficiation
Sel 44,153 particles
Auto-Refinement
Report 4.3 Å
Masked Refine 3D
Report 3.5 Å
c
Final resolution = 3.5 Angstroms
Fourier Shell Correlation
resolution (1/Å)
3.5 Å
d
Final resolution = 3.5 Angstroms
Fourier Shell Correlation
resolution (1/Å)
3.5 Å
e
resolution (Å)
FSC
1/resolution (1/Å)
3.7 Å
FSC
FSCsum
FSCwork
FSCfree
f
resolution (Å)
FSC
1/resolution (1/Å)
3.8 Å
FSC
FSCsum
FSCwork
FSCfree

Supplementary Figure 2. Cryo-EM data processing of the apo ClC-2 and its complex with AK-42. Datasets for the ClC-2 apo form (**a**) and the AK-42 bound form (**b**) were imported into Relion for motion correction and Contrast Transfer Function (CTF) estimation. For apo state ClC-2, total 7374 micrographs were used. For AK-42 bound ClC-2, total 8951 micrographs were used. ClC-2 particles were auto-picked, extracted, and further subjected to 2D classification. Good 2D classes were selected and imported into cryoSPARC for initial model building and 3D classification. Non-uniform refinement and masked refinement were performed to improve the resolution of the final maps. The resolutions of the final refined maps were determined according to Fourier shell correlation (FSC) with a criterion of 0.143, the angular distributions were also shown (**c, d**). Final models were validated to prevent overfitting using the FSC between map and model with a criterion of 0.5 (**e, f**).

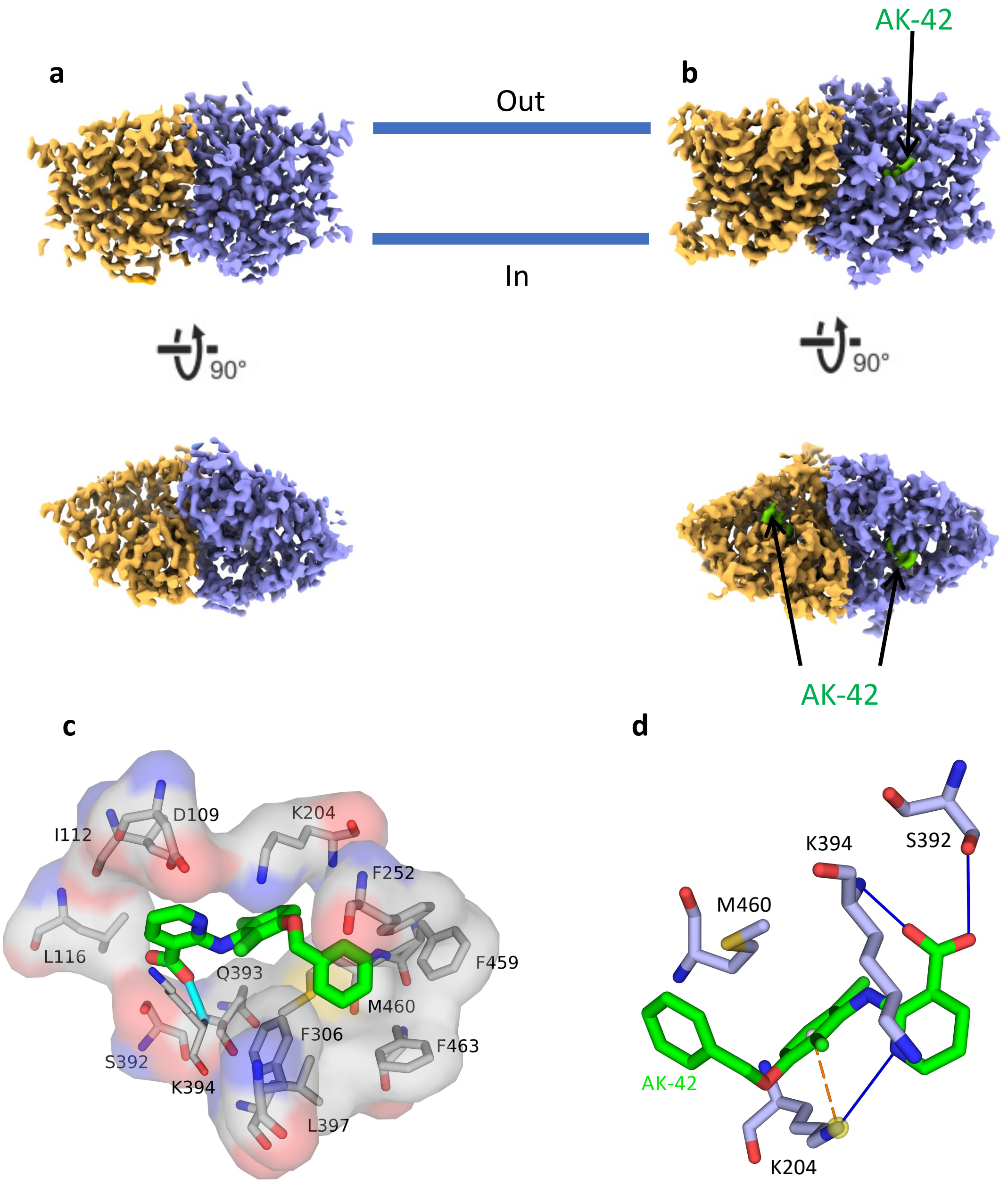

Supplementary Figure 3. Cryo-EM maps of the TMD domains of the apo ClC-2 and its complex with AK-42. (**a**) The front view and top view of the masked refined apo ClC-2 transmembrane domain (TMD). (**b**) The front view and top view of masked refined AK-42 (green density) bound ClC-2 TMD domain. Protomers are shown in brown and purple, respectively. (**c**) Surrounding residues around AK-42 inhibitor. AK-42 is shown in green. Surrounding residues are shown in stick and surface representation. Blue indicates positive potential and red indicates negative charge. (**d**) Structural analysis using PLIP (https://plip-tool.biotec.tu-dresden.de/plip-web/plip/index) identified potentials hydrogen bonds and π-cation interaction between AK-42 and K204, S392, K394. Blue lines represent hydrogen bonds. Brown dashed line represents π-cation interaction.

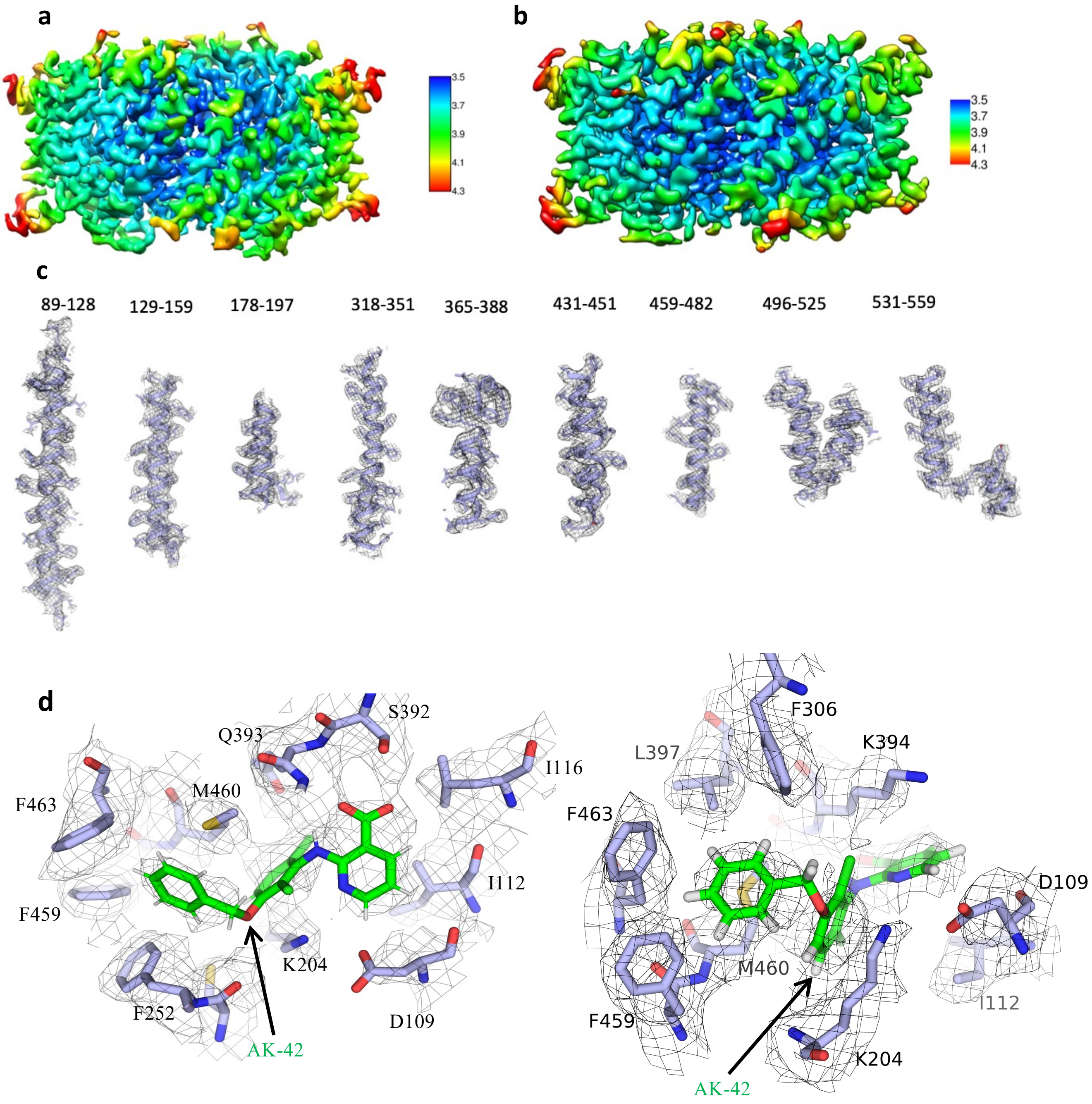

Supplementary Figure 4. Local resolution of ClC-2 cryo-EM maps. Local resolution values were calculated in RELION using the relion_postprocess module, and the colored density maps were displayed in UCSF Chimera. Apo ClC-2 (**a**) and AK-42 bound ClC-2 (**b**) are shown. (**c**) Selected regions are shown to demonstrate the good fit between the model and maps of apo ClC-2. (**d**) Density of AK-42 and surrounding residues to show the map quality of AK-42 bound ClC-2. Two different views are shown.

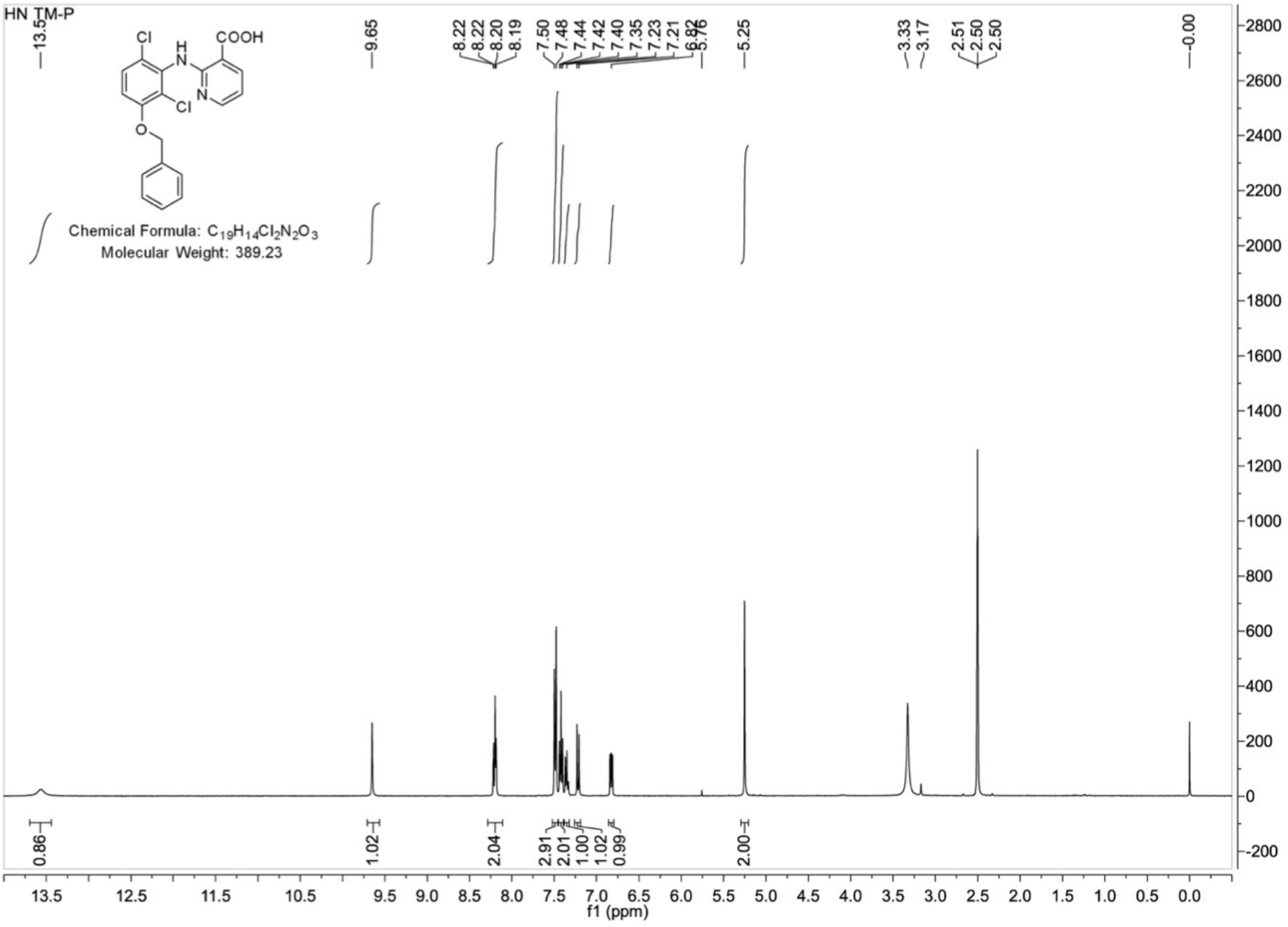

Supplementary Figure 5. Synthesis and confirmation of AK-42 by ^{1}H-NMR spectroscopy. The specific inhibitor AK-42 was synthesized as described in the section "*Chemical synthesis of AK-42*". The product was confirmed by ^{1}H-NMR.

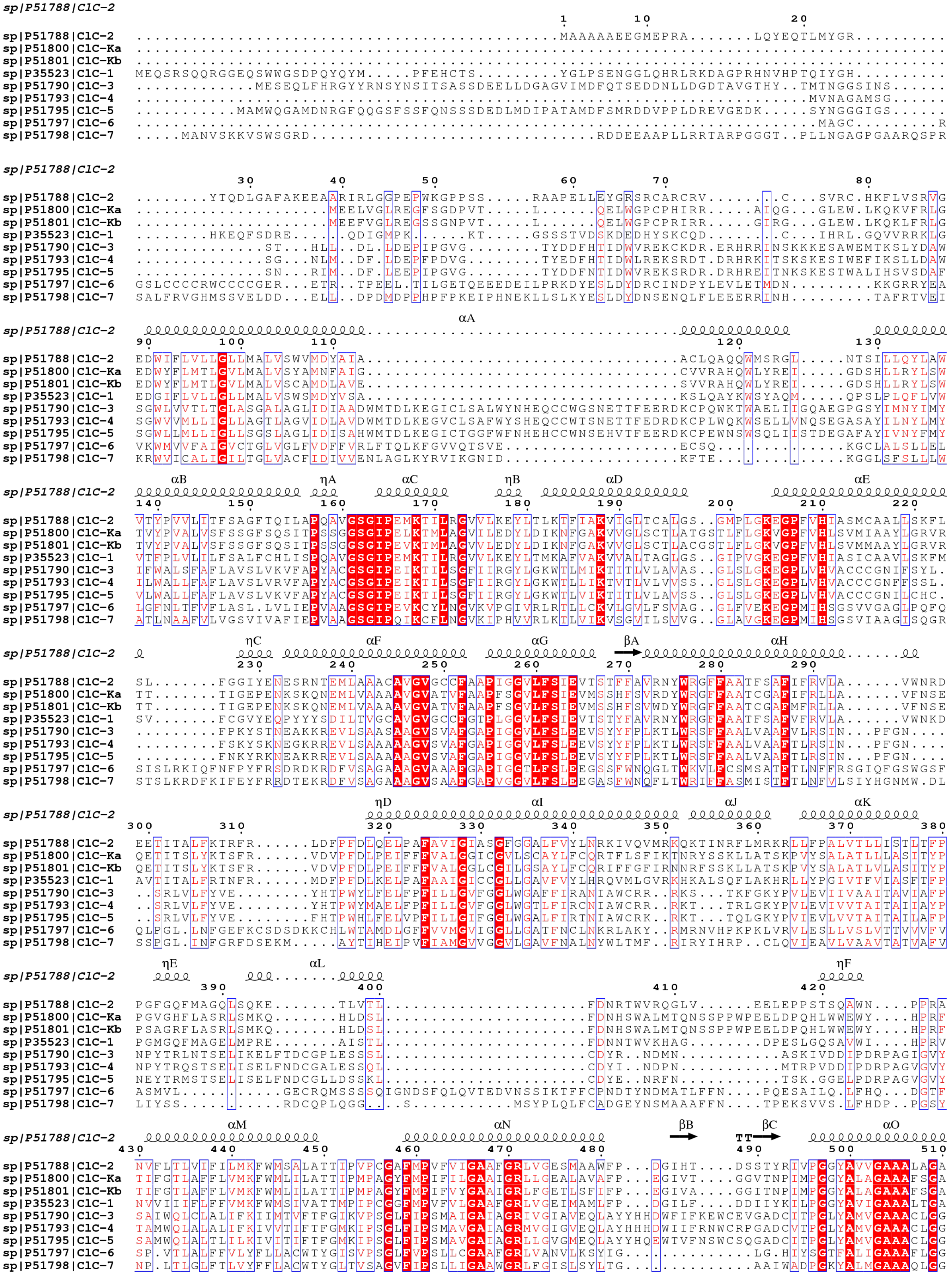
sp|P51788|ClC-2
sp|P51788|ClC-2 ..............................MAAAAAEEGMEPRA.......LQYEQTLMYGR.........
sp|P51800|ClC-Ka ....
sp|P51801|ClC-Kb ....
sp|P35523|ClC-1 MEQSRSQQRGGEQSWWGSDPQYQYM.....PFEHCTS.........YGLPSENGGLQHRLRKDAGPRHNVHPTQIYGH..........
sp|P51790|ClC-3 .............MESEQLFHRGYYRNSYNSITSASSDEELLDGAGVIMDFQTSEDDNLLDGDTAVGTHY..TMTNGGSINS......
sp|P51793|ClC-4 ....MVNAGAMSG......
sp|P51795|ClC-5 .........MAMWQGAMDNRGFQQGSFSSFQNSSSDEDLMDIPATAMDFSMRDDVPPLDREVGEDK.....SYNGGGIGS......
sp|P51797|ClC-6 ....MAGC.........R
sp|P51798|ClC-7 .....MANVSKKVSWSGRD.............................RDDEEAAPLLRRTARPGGGT..PLLNGAGPGAARQSPR
sp|P51788|ClC-2 ........YTQDLGAFAKEEAARIRLGGPEPWKGPPSS.....RAAPELLEYGRSRCARCRV.......C...SVRC.HKFLVSRVG
sp|P51800|ClC-Ka ....................MEELVGLREGFSGDPVT.....L......QELWGPCPHIRR.....AIQG...GLEW.LKQKVFRLG
sp|P51801|ClC-Kb ....................MEEFVGLREGSSGNPVT.....L......QELWGPCPRIRR.....GIRG...GLEW.LKQKLFRLG
sp|P35523|ClC-1 ........HKEQFSDRE.....QDIGMPK.....KT.....GSSSTVDSKDEDHYSKCQD.......C...IHRL.GQVVRRKLG
sp|P51790|ClC-3 ..............ST...HLL..DL.LDEPIPGVG.......TYDDFHTIDWVREKCKDR.ERHRRINSKKKESAWEMTKSLYDAW
sp|P51793|ClC-4 ..............SG...NLM..DF.LDEPFPDVG.......TYEDFHTIDWLREKSRDT.DRHRKITSKSKESIWEFIKSLLDAW
sp|P51795|ClC-5 ..............SN...RIM..DF.LEEPIPGVG.......TYDDFNTIDWVREKSRDR.DRHREITNKSKESTWALIHSVSDAF
sp|P51797|ClC-6 GSLCCCCRWCCCCGER...ETR..TPEEL.TILGETQEEEDEILPRKDYESLDYDRCINDPYLEVLETMDN.........KKGRRYEA
sp|P51798|ClC-7 SALFRVGHMSSVELDD...ELL..DPDMDPHPFPKEIPHNEKLLSLKYESLDYDNSENQLFLEEERRINH.........TAFRTVEI
αA
sp|P51788|ClC-2 EDWIFLVLLGLLMALVSWVMDYAIA...........................ACLQAQQWMSRGL.....NTSILLQYLAW
sp|P51800|ClC-Ka EDWYFLMTLGVLMALVSYAMNFAIG...........................CVVRAHQWLYREI.....GDSHLLRYLSW
sp|P51801|ClC-Kb EDWYFLMTLGVLMALVSCAMDLAVE...........................SVVRAHQWLYREI.....GDSHLLRYLSW
sp|P35523|ClC-1 EDGIFLVLLGLLMALVSWSMDYVSA...........................KSLQAYKWSYAQM.....QPSLPLQFLVW
sp|P51790|ClC-3 SGWLVVTLTGLASGALAGLIDIAADWMTDLKEGICLSALWYNHEQCCWGSNETTFEERDKCPQWKTWAELIIGQAEGPGSYIMNYIMY
sp|P51793|ClC-4 SGWVVMLLIGLLAGTLAGVIDLAVDWMTDLKEGVCLSAFWYSHEQCCWTSNETTFEDRDKCPLWQKWSELLVNQSEGASAYILNYLMY
sp|P51795|ClC-5 SGWLLMLLIGLLSGSLAGLIDISAHWMTDLKEGICTGGFWFNHEHCCWNSEHVTFEERDKCPEWNSWSQLIISTDEGAFAYIVNYFMY
sp|P51797|ClC-6 VKWMVVFAIGVCTGLVGLFVDFFVRLFTQLKFGVVQTSVE...........ECSQ...........KGCLALSLLEL
sp|P51798|ClC-7 KRWVICALIGILTGLVACFIDIVVENLAGLKYRVIKGNID...........KFTE...........KGGLSFSLLLW
αB ηA αC ηB αD αE
sp|P51788|ClC-2 VTYPVVLITFSAGFTQILAPQAVGSGIPEMKTILRGVVLKEYLTLKTFIAKVIGLTCALGS..GMPLGKEGPFVHIASMCAALLSKFL
sp|P51800|ClC-Ka TVYPVALVSFSSGFSQSITPSSGGSGIPELKTMLAGVILEDYLDIKNFGAKVVGLSCTLATGSTLFLGKVGPFVHLSVMIAAYLGRVR
sp|P51801|ClC-Kb TVYPVALVSFSSGFSQSITPSSGGSGIPEVKTMLAGVVLEDYLDIKNFGAKVVGLSCTLACGSTLFLGKVGPFVHLSVMMAAYLGRVR
sp|P35523|ClC-1 VTFPLVLILFSALFCHLISPQAVGSGIPEMKTILRGVVLKEYLTMKAFVAKVVALTAGLGS..GIPVGKEGPFVHIASICAAVLSKFM
sp|P51790|ClC-3 IFWALSFAFLAVSLVKVFAPYACGSGIPEIKTILSGFIIRGYLGKWTLMIKTITLVLAVAS..GLSLGKEGPLVHVACCCGNIFSYL.
sp|P51793|ClC-4 ILWALLFAFLAVSLVRVFAPYACGSGIPEIKTILSGFIIRGYLGKWTLLIKTVTLVLVVSS..GLSLGKEGPLVHVACCCGNFFSSL.
sp|P51795|ClC-5 VLWALLFAFLAVSLVKVFAPYACGSGIPEIKTILSGFIIRGYLGKWTLVIKTITLVLAVSS..GLSLGKEGPLVHVACCCGNILCHC.
sp|P51797|ClC-6 LGFNLTFVFLASL.LVLIEPVAAGSGIPEVKCYLNGVKVPGIVRLRTLLCKVLGVLFSVAG..GLFVEKEGPMIHSGSVVGAGLPQFQ
sp|P51798|ClC-7 ATLNAAFVLVGSVIVAFIEPVAAGSGIPQIKCFLNGVKIPHVVRLKTLVIKVSGVILSVVG..GLAVGKEGPMIHSGSVIAAGISQGR
ηC αF αG βA αH
sp|P51788|ClC-2 SL.......FGGIYENESRNTEMLAAACAVGVGCCFAAPIGGVLFSIEVTSTFFAVRNYWRGFFAATFSAFIFRVLA......VWNRD
sp|P51800|ClC-Ka TT.......TIGEPENKSKQNEMLVAAAAVGVATVFAAPFSGVLFSIEVMSSHFSVRDYWRGFFAATCGAFIFRLLA......VFNSE
sp|P51801|ClC-Kb TT.......TIGEPENKSKQNEMLVAAAAVGVATVFAAPFSGVLFSIEVMSSHFSVWDYWRGFFAATCGAFMFRLLA......VFNSE
sp|P35523|ClC-1 SV.......FCGVYEQPYYYSDILTVGCAVGVGCCFGTPLGGVLFSIEVTSTYFAVRNYWRGFFAATFSAFVFRVLA......VWNKD
sp|P51790|ClC-3 .........FPKYSTNEAKKREVLSAASAAGVSVAFGAPIGGVLFSLEEVSYYFPLKTLWRSFFAALVAAFVLRSIN...PFGN....
sp|P51793|ClC-4 .........FSKYSKNEGKRREVLSAAAAAGVSVAFGAPIGGVLFSLEEVSYYFPLKTLWRSFFAALVAAFTLRSIN...PFGN....
sp|P51795|ClC-5 .........FNKYRKNEAKRREVLSAAAAAGVSVAFGAPIGGVLFSLEEVSYYFPLKTLWRSFFAALVAAFTLRSIN...PFGN....
sp|P51797|ClC-6 SISLRKIQFNFPYFRSDRDKRDFVSAGAAAGVAAAFGAPIGGTLFSLEEGSSFWNQGLTWKVLFCSMSATFTLNFFRSGIQFGSWGSF
sp|P51798|ClC-7 STSLKRDFKIFEYFRRDTEKRDFVSAGAAAGVSAAFGAPVGGVLFSLEEGASFWNQFLTWRIFFASMISTFTLNFVLSIYHGNMW.DL
ηD αI αJ αK
sp|P51788|ClC-2 EETITALFKTRFR......LDFPFDLQELPAFAVIGIASGFGGALFVYLNRKIVQVMRKQKTINRFLMRKRLLFPALVTLLISTLTFP
sp|P51800|ClC-Ka QETITSLYKTSFR......VDVPFDLPEIFFFVALGGICGVLSCAYLFCQRTFLSFIKTNRYSSKLLATSKPVYSALATLLLASITYP
sp|P51801|ClC-Kb QETITSLYKTSFR......VDVPFDLPEIFFFVALGGLCGILGSAYLFCQRIFFGFIRNNRFSSKLLATSKPVYSALATLVLASITYP
sp|P35523|ClC-1 AVTITALFRTNFR......MDFPFDLKELPAFAAIGICCGLLGAVFVYLHRQVMLGVRKHKALSQFLAKHRLLYPGIVTFVIASFTFP
sp|P51790|ClC-3 ..SRLVLFYVE.......YHTPWYLFELFPFILLGVFGGLWGAFFIRANIAWCRR..RKS...TKFGKYPVLEVIIVAAITAVIAFP
sp|P51793|ClC-4 ..SRLVLFYVE.......YHTPWYMAELFPFILLGVFGGLWGTLFIRCNIAWCRR..RKT...TRLGKYPVLEVIVVTAITAIIAYP
sp|P51795|ClC-5 ..SRLVLFYVE.......FHTPWHLFELVPFILLGIFGGLWGALFIRTNIAWCRK..RKT...TQLGKYPVIEVLVVTAITAILAFP
sp|P51797|ClC-6 QLPGL.LNFGEFKCSDSDKKCHLWTAMDLGFFVVMGVIGGLLGATFNCLNKRLAKY..RMRNVHPKPKLVRVLESLLVSLVTTVVVFV
sp|P51798|ClC-7 SSPGL.INFGRFDSEKM.....AYTIHEIPVFIAMGVVGGVLGAVFNALNYWLTMF..RIRYIHRP..CLQVIEAVLVAAVTATVAFV
ηE αL ηF
sp|P51788|ClC-2 PGFGQFMAGQLSQKE.......TLVTL........................FDNRTWVRQGLV.......EELEPPSTSQAWN....PPRA
sp|P51800|ClC-Ka PGVGHFLASRLSMKQ.......HLDSL........................FDNHSWALMTQNSSPPWPEELDPQHLWWEWY....HPRF
sp|P51801|ClC-Kb PSAGRFLASRLSMKQ.......HLDSL........................FDNHSWALMTQNSSPPWPEELDPQHLWWEWY....HPRF
sp|P35523|ClC-1 PGMGQFMAGELMPRE.......AISTL........................FDNNTWVKHAG.......DPESLGQSAVWI....HPRV
sp|P51790|ClC-3 NPYTRLNTSELIKELFTDCGPLESSSL........................CDYR..NDMN...........ASKIVDDIPDRPAGIGVY
sp|P51793|ClC-4 NPYTRQSTSELISELFNDCGALESSQL........................CDYI..NDPN...........MTRPVDDIPDRPAGVGVY
sp|P51795|ClC-5 NEYTRMSTSELISELFNDCGLLDSSKL........................CDYE..NRFN...........TSK.GGELPDRPAGVGVY
sp|P51797|ClC-6 ASMVL.........GECRQMSSSSQIGNDSFQLQVTEDVNSSIKTFFCPNDTYNDMATLFFN....PQESAILQ.LFHQ...DGTF
sp|P51798|ClC-7 LIYSS...........RDCQPLQGG..S...........MSYPLQLFCADGEYNSMAAAFFN....TPEKSVVS.LFHDP..PGSY
αM αN βB TT βC αO
sp|P51788|ClC-2 NVFLTLVIFILMKFWMSALATTIPVPCGAFMPVFVIGAAFGRLVGESMAAWFP...DGIHT....DSSTYRIVPGGYAVVGAAALAGA
sp|P51800|ClC-Ka TIFGTLAFFLVMKFWMLILATTIPMPAGYFMPIFILGAAIGRLLGEALAVAFP...EGIVT....GGVTNPIMPGGYALAGAAAFSGA
sp|P51801|ClC-Kb TIFGTLAFFLVMKFWMLILATTIPMPAGYFMPIFVYGAAIGRLFGETLSFIFP...EGIVA....GGITNPIMPGGYALAGAAAFSGA
sp|P35523|ClC-1 NVIIIFLFFVMKFWMSIVATTMPIPCGGFMPVFVLGAAFGRLVGEIMAMLFP...DGILF....DDIIYKILPGGYAVIGAAALTGA
sp|P51790|ClC-3 SAIWQLCLALIFKIIMTVFTFGIKVPSGLFIPSMAIGAIAGRIVGIAVEQLAYYHHDWFIFKEWCEVGADCITPGLYAMVGAAACLGG
sp|P51793|ClC-4 TAMWQLALALIFKIVVTIFTFGMKIPSGLFIPSMAVGAIAGRMVGIGVEQLAYHHHDWIIFRNWCRPGADCVTPGLYAMVGAAACLGG
sp|P51795|ClC-5 SAMWQLALTLILKIVITIFTFGMKIPSGLFIPSMAVGAIAGRLLGVGMEQLAYYHQEWTVFNSWCSQGADCITPGLYAMVGAAACLGG
sp|P51797|ClC-6 SP.VTLALFFVLYFLLACWTYGISVPSGLFVPSLLCGAAFGRLVANVLKSYIG..............LG.HIYSGTFALIGAAAFLGG
sp|P51798|ClC-7 NP.LTLGLFTLVYFFLACWTYGLTVSAGVFIPSLLIGAAWGRLFGISLSYLTG...........AAIWADPGKYALMGAAAQLGG

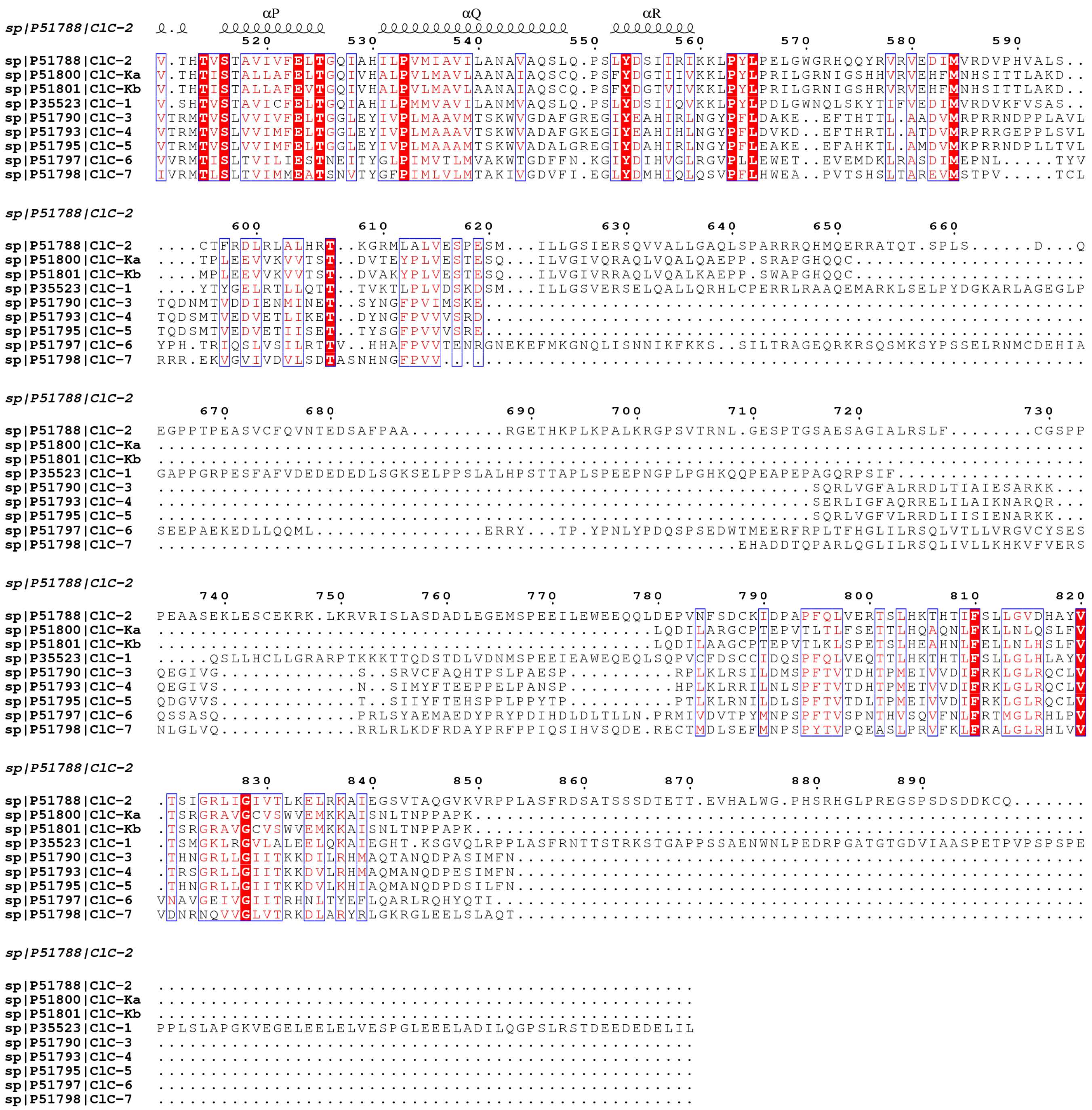

Supplementary Figure 6. Sequence alignment of human ClC family proteins. Human ClC-2, ClC-Ka, ClC-Kb, ClC-1, ClC-5, ClC-3, ClC-4, ClC-6 and ClC-7 were used for alignment. Conserved residues are indicated by blue boxes, and identical residues are highlighted in red. The secondary structure of ClC-2 is shown as a reference above the sequence alignment. 18 helices of human ClC-2 are labeled as αA to αR respectively.

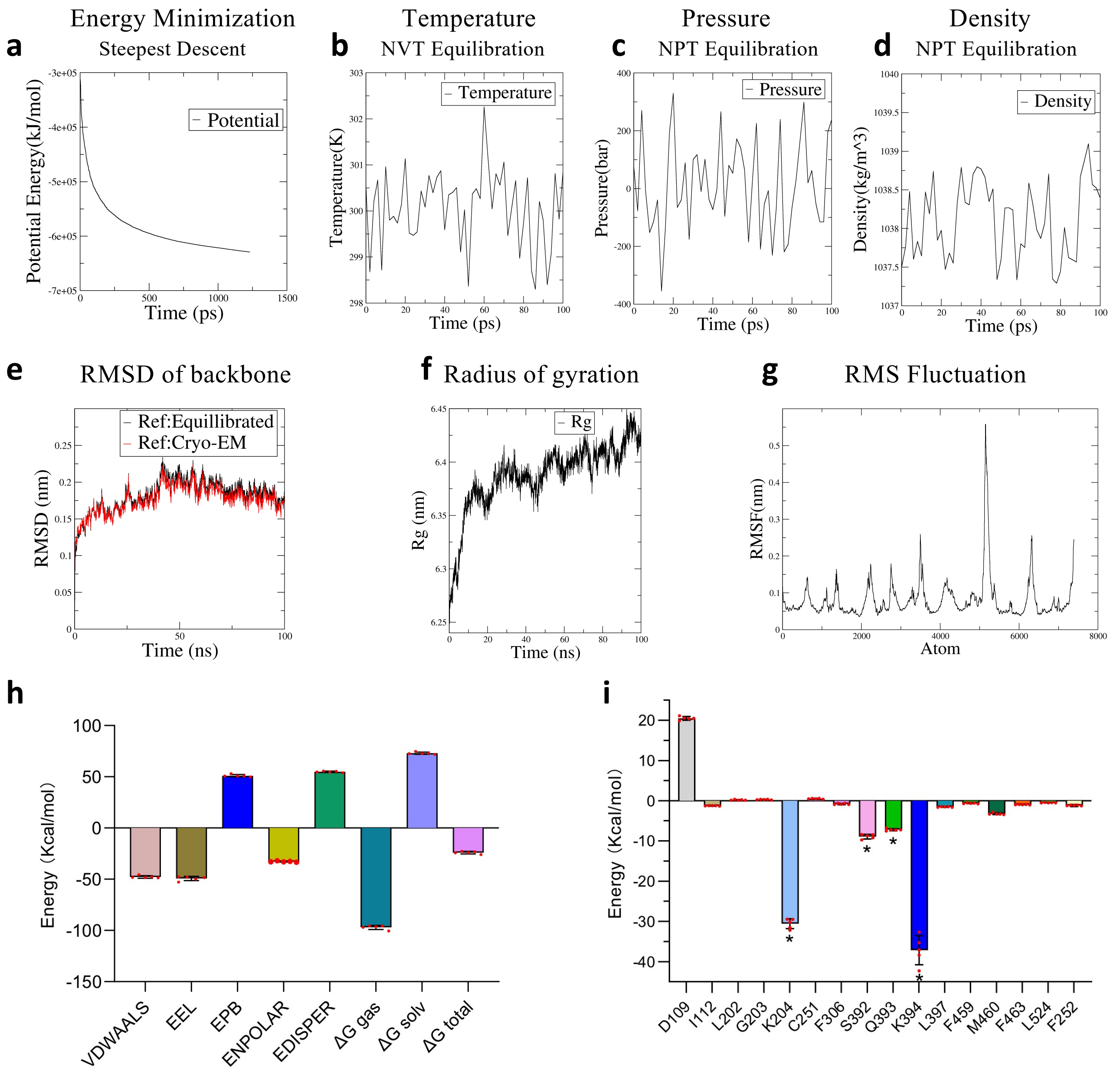

Supplementary Figure 7. Molecular dynamics simulations of ClC-2 bound with AK-42. Energy minimization (**a**), *NVT* equilibration (**b**), and *NPT* equilibration (**c, d**) were performed before the running of molecular dynamics simulations. The root-mean-square deviation (RMSD) (**e**), radius of gyration (**f**), and root mean square fluctuation (RMSF) (**g**) were analyzed after simulations. (**h**) The energy components for the interaction between ClC-2 and AK-42 were analyzed. (**i**) Per-residue energy decomposition of the residues in the ClC-2/AK-42 interface using the MM/PBSA method. * indicates the three most critical residues in the binding with AK-42. VDWAALS, van der Waals energy. EEL, electrostatic energy. ENPOLAR, nonpolar solvation energy. EDISPER, dispersion energy. ΔG_{gas}, relative gas-phase Gibbs free energy. ΔG_{solv}, solvation free energy. Source data are provided as a Source Data file. Bars in **h** and **i** indicate means ± SEM (n = 5 independent MD runs used for MM/PBSA calculation).

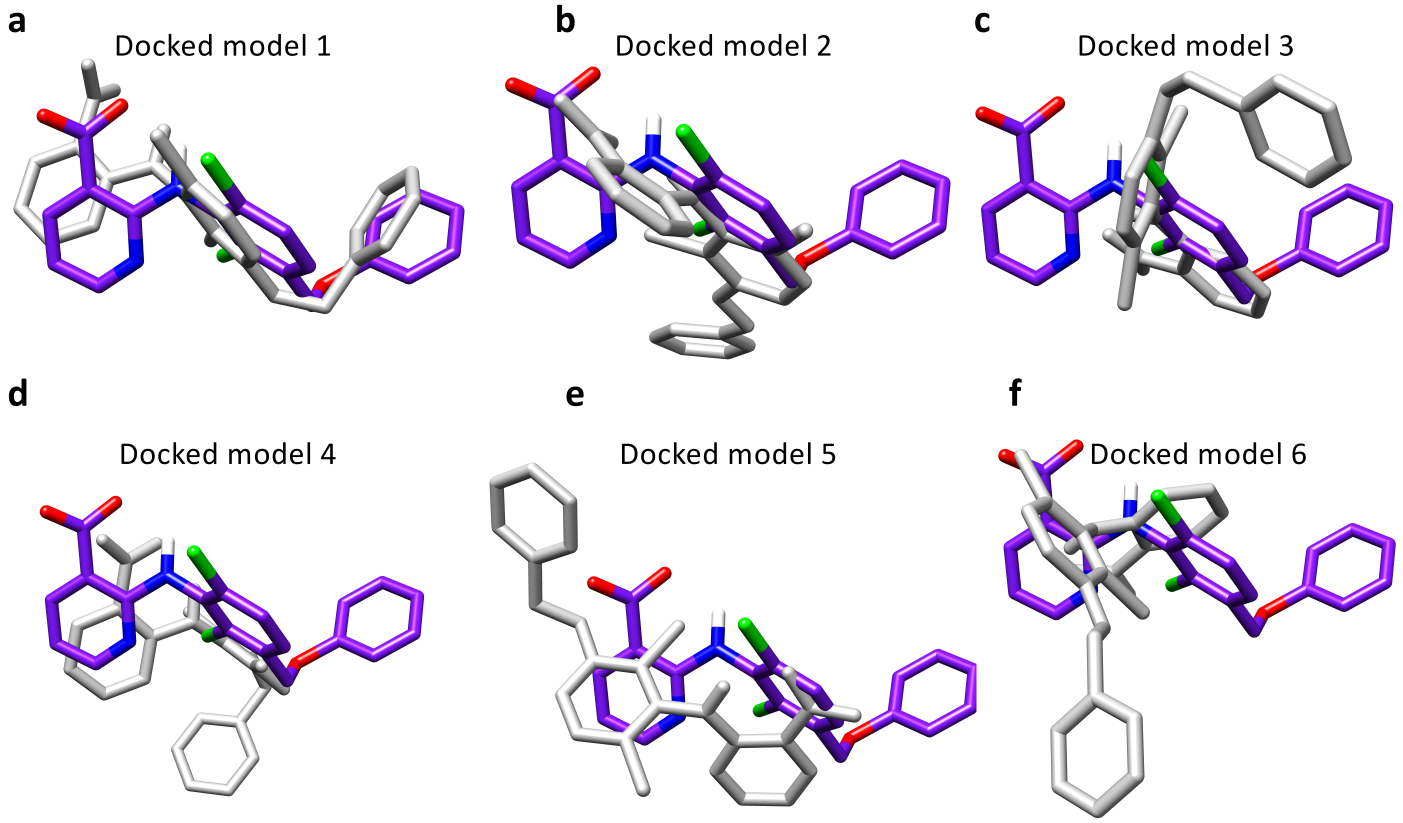

g

Key residues	D109	G203	K204	F306	S392	Q393	K394	M460	F463	L524
Cryo-EM model	√		√	√	√	√	√	√	√	√
Docked model 1		√	√		√	√	√	√		
Docked model 2			√		√	√	√	√		√
Docked model 3		√	√			√	√	√		√
Docked model 4	√		√		√	√	√	√		
Docked model 5	√	√	√		√			√		
Docked model 6	√									

Supplementary Figure 8. Structural comparison between AK-42 from cryo-EM model and previous docked models. ClC-2 structures from cryo-EM and previous docked models[1] are aligned. AK-42 from cryo-EM model was shown in purple color. AK-42 from docked models is shown in gray color. ClC-2 models were not shown for simplification. Panel **(a-f)** demonstrate structural alignment between cryo-EM model with 6 docked models with top scores in the previous report. **(g)** Key residues confirmed by electrophysiological recordings in our studies were extracted and listed in the first row. For each model from cryo-EM models or docked models, tick mark "√" under each residue indicate that this residue was identified by the specific model. Numbering for residues is according to human ClC-2. For D109, G203, K204, F306, S392, Q393, K394, M460, F463 and L524 from human ClC-2, the corresponding residues from docked rat ClC-2 models are D115, G209, K210, F312, S398, Q399, K400, M466, F469 and L530, respectively.

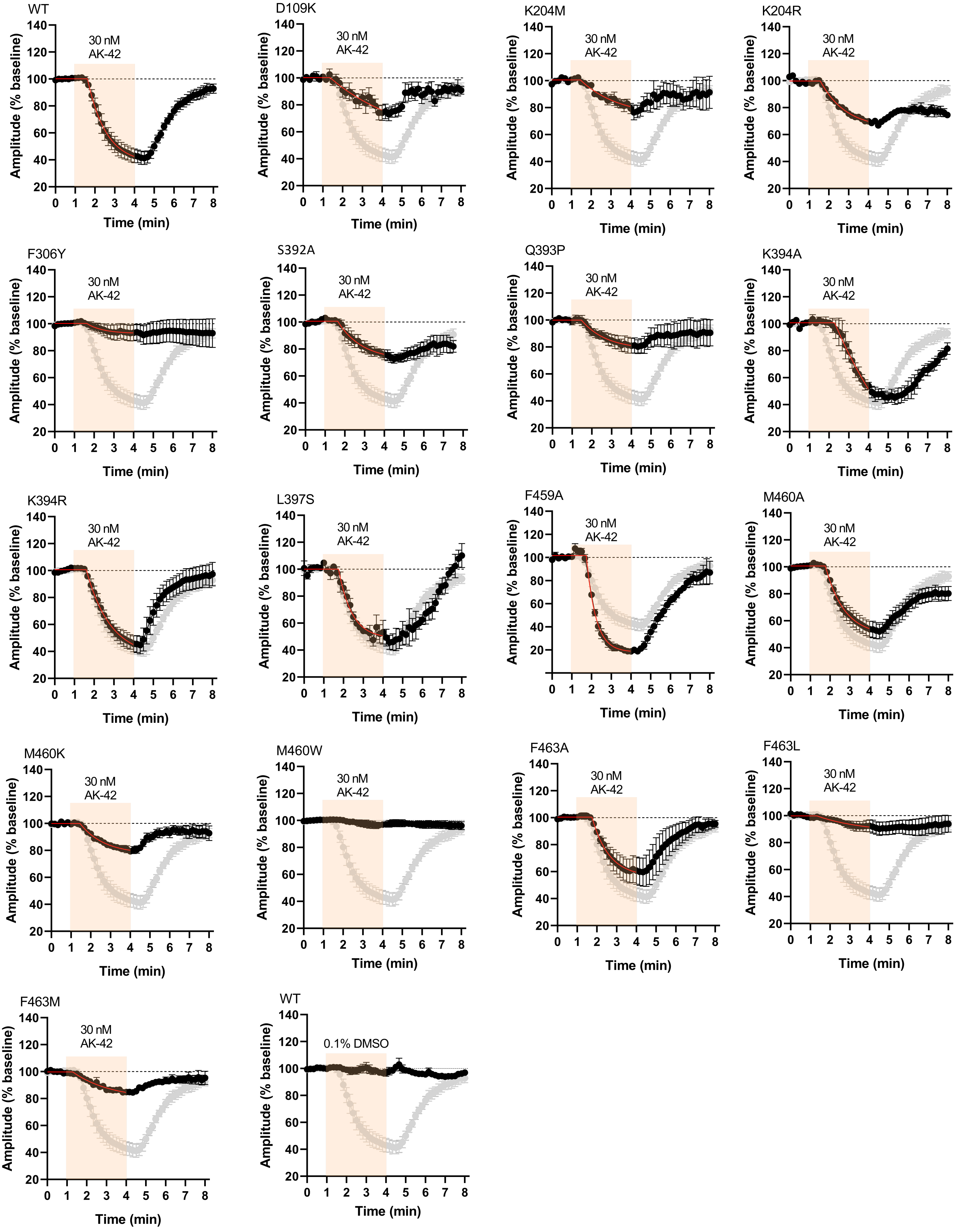
WT
D109K
K204M
K204R
F306Y
S392A
Q393P
K394A
K394R
L397S
F459A
M460A
M460K
M460W
F463A
F463L
F463M
WT
30 nM
AK-42
0.1% DMSO
Amplitude (% baseline)
Time (min)

Supplementary Figure 9. Inhibition kinetics of AK-42 with a time course for wild-type ClC-2 and its mutants. The onset of AK-42 block was demonstrated by recording the current traces with a time course of 8 min. The baseline was recorded in 0-1 min just before the application of 30 nM AK-42. Then currents during the application of AK-42 were recorded in 1-4 min, as indicated by brown color. Currents during 4-8 min represent the washout of AK-42 inhibitor. For the data of each mutation in black color, the data of wild-type (WT) ClC-2 was shown in gray color for comparison. Red curves represent the fitting curves of the experiment data based on Supplementary Table 2. Total number of cells used for wild-type ClC-2 and each mutant can be found in Supplementary Table 2.

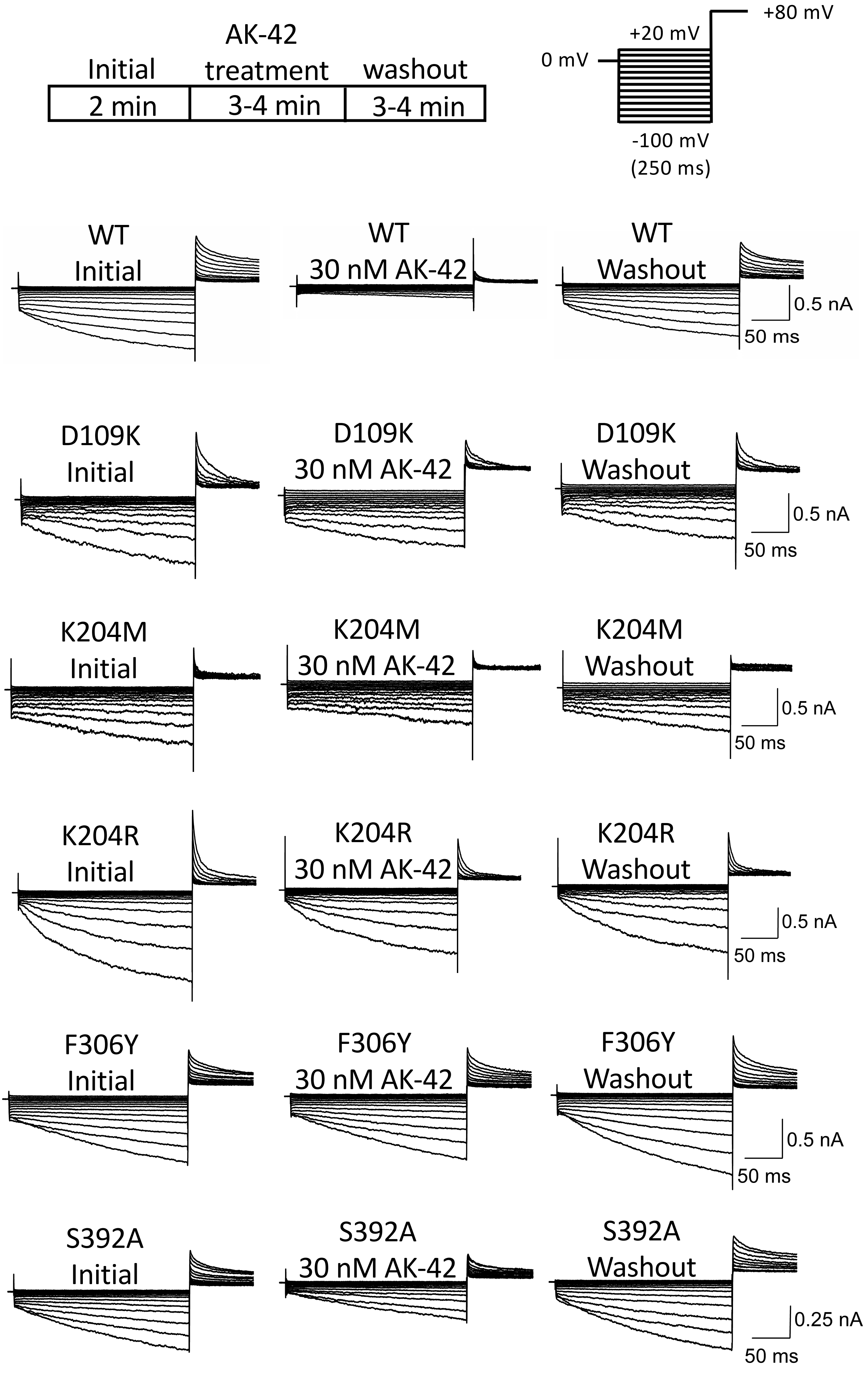

AK-42
Initial
treatment
washout
2 min
3-4 min
3-4 min
+80 mV
+20 mV
0 mV
-100 mV
(250 ms)
WT
Initial
WT
30 nM AK-42
WT
Washout
0.5 nA
50 ms
D109K
Initial
D109K
30 nM AK-42
D109K
Washout
0.5 nA
50 ms
K204M
Initial
K204M
30 nM AK-42
K204M
Washout
0.5 nA
50 ms
K204R
Initial
K204R
30 nM AK-42
K204R
Washout
0.5 nA
50 ms
F306Y
Initial
F306Y
30 nM AK-42
F306Y
Washout
0.5 nA
50 ms
S392A
Initial
S392A
30 nM AK-42
S392A
Washout
0.25 nA
50 ms

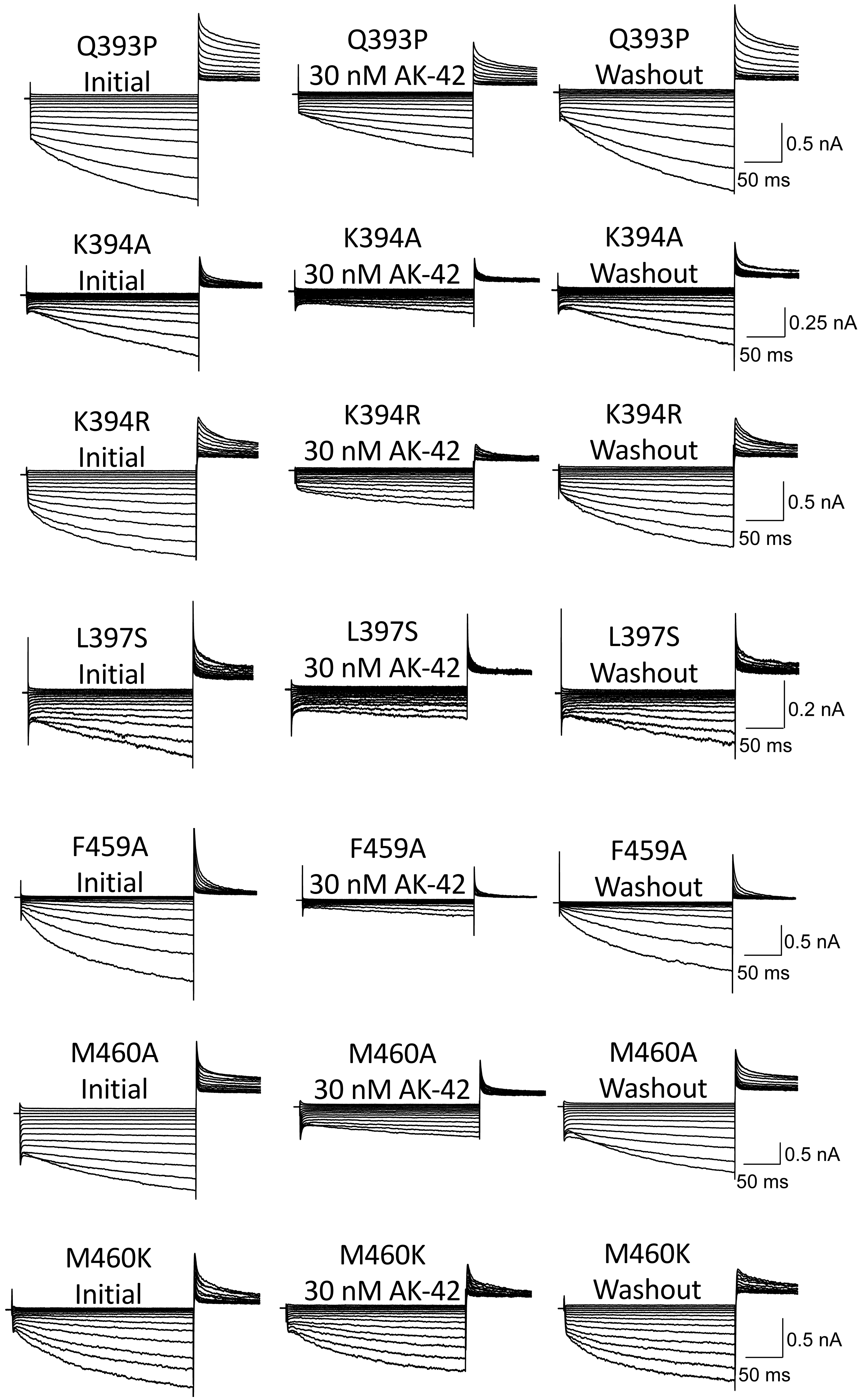
Q393P
Initial
Q393P
30 nM AK-42
Q393P
Washout
0.5 nA
50 ms
K394A
Initial
K394A
30 nM AK-42
K394A
Washout
0.25 nA
50 ms
K394R
Initial
K394R
30 nM AK-42
K394R
Washout
0.5 nA
50 ms
L397S
Initial
L397S
30 nM AK-42
L397S
Washout
0.2 nA
50 ms
F459A
Initial
F459A
30 nM AK-42
F459A
Washout
0.5 nA
50 ms
M460A
Initial
M460A
30 nM AK-42
M460A
Washout
0.5 nA
50 ms
M460K
Initial
M460K
30 nM AK-42
M460K
Washout
0.5 nA
50 ms

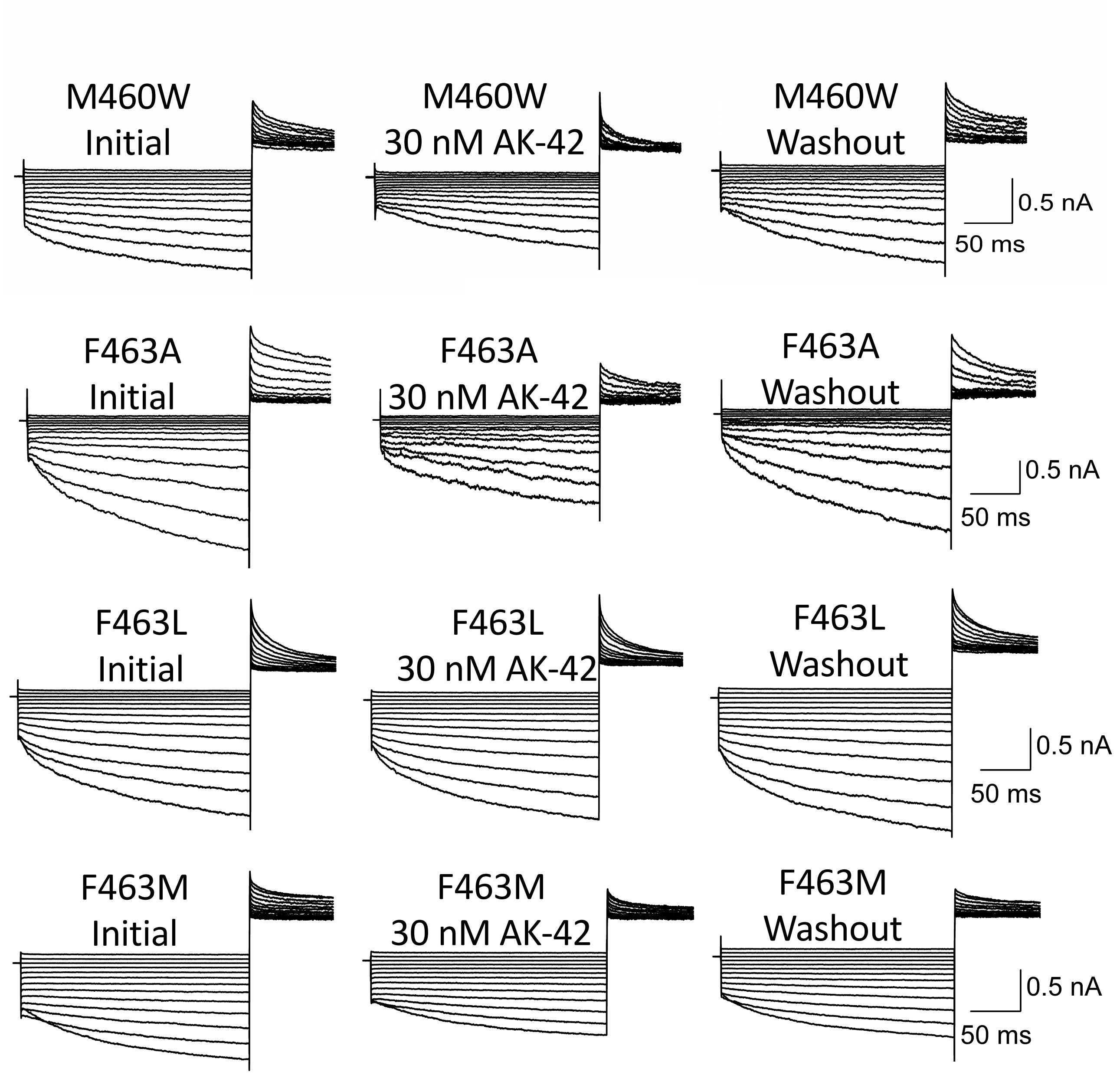

Supplementary Figure 10. Current traces for wild-type ClC-2 and its mutants. ClC-2 plasmids were transfected into CHO-K1 cells and whole-cell patch clamp experiments were performed to record currents across the membrane. For wild-type ClC-2 and each mutant, current traces before AK-42 treatment (initial), after treatment (30 nM AK-42) and after AK-42 washout (washout) were shown. Detailed protocol can be found in the Method section. Total number of cells used for wild-type ClC-2 and each mutant can be found in Supplementary Table 3.

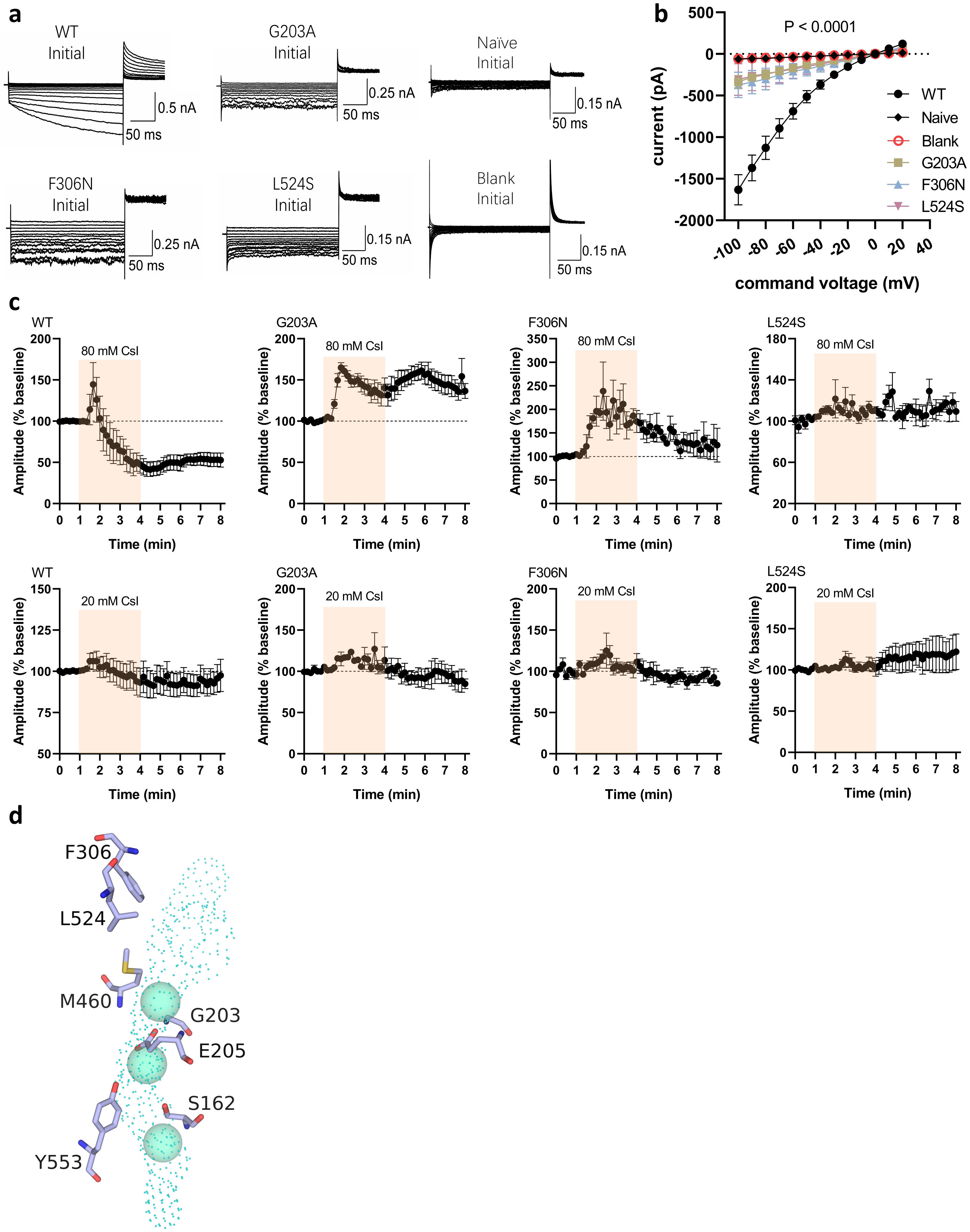
a
WT Initial
0.5 nA
50 ms
G203A Initial
0.25 nA
50 ms
Naïve Initial
0.15 nA
50 ms
F306N Initial
0.25 nA
50 ms
L524S Initial
0.15 nA
50 ms
Blank Initial
0.15 nA
50 ms
b
P < 0.0001
current (pA)
command voltage (mV)
WT
Naive
Blank
G203A
F306N
L524S
c
WT
G203A
F306N
L524S
80 mM CsI
20 mM CsI
Amplitude (% baseline)
Time (min)
d
F306
L524
M460
G203
E205
S162
Y553

Supplementary Figure 11. Inhibition of hyperpolarization-induced currents in CHO-K1 cells transfected with ClC-2 wild-type or mutation plasmids. (**a**) Representative current traces of ClC-2 WT, G203A, F306N, L524S, un-transfected controls (Naive) and empty-vector transfected control (Blank) without AK-42 treatment (initial). (**b**) Whole-cell current-voltage (I-V) curves differed significantly among ClC-2 WT, G203A, F306N, L524S, un-transfected controls (Naive) and empty-vector transfected controls (Blank). N values represent biologically independent cells recorded. (n = 14 for WT, n = 5 for G203A and F306N, n = 3 for L524S, n = 3 for Naive, n = 3 for Blank. Two-way ANOVA with Dunnett's multiple comparisons test. Factor of command voltage x mutations, $F (60,324) = 10.78$, $P < 0.0001$; factor of command voltage, $F (1.056, 28.52) = 19.26$, $P = 0.0001$; factor of mutations, $F (5, 27) = 9.255$, $P < 0.0001$). Data are presented as the mean ± SEM. Source data are provided as a Source Data file. (**c**) Current traces with application of cesium iodide (CsI) in the who-cell patch clamp experiments. 80 mM and 20 mM CsI were used for wild-type ClC-2 and G203A, F306N and L524S mutants, respectively. CsI was applied during 1-4 min in the time course, as indicated by brown color. (n = 6 for WT + 80 mM CsI, n = 5 for WT + 20 mM CsI, n = 6 for G203A + 80 mM CsI, n = 3 for G203A + 20 mM CsI, n = 4 for F306N + 80 mM CsI, n = 4 for F306N + 20 mM CsI, n =3 for L524S + 80 mM CsI, n = 4 for L524S + 20 mM CsI). Data are presented as the mean ± SEM. (**d**) Structure analysis shows that G203, F306N and L524S are close to the ClC-2 channel and might be involved in the channel regulation of ClC-2.

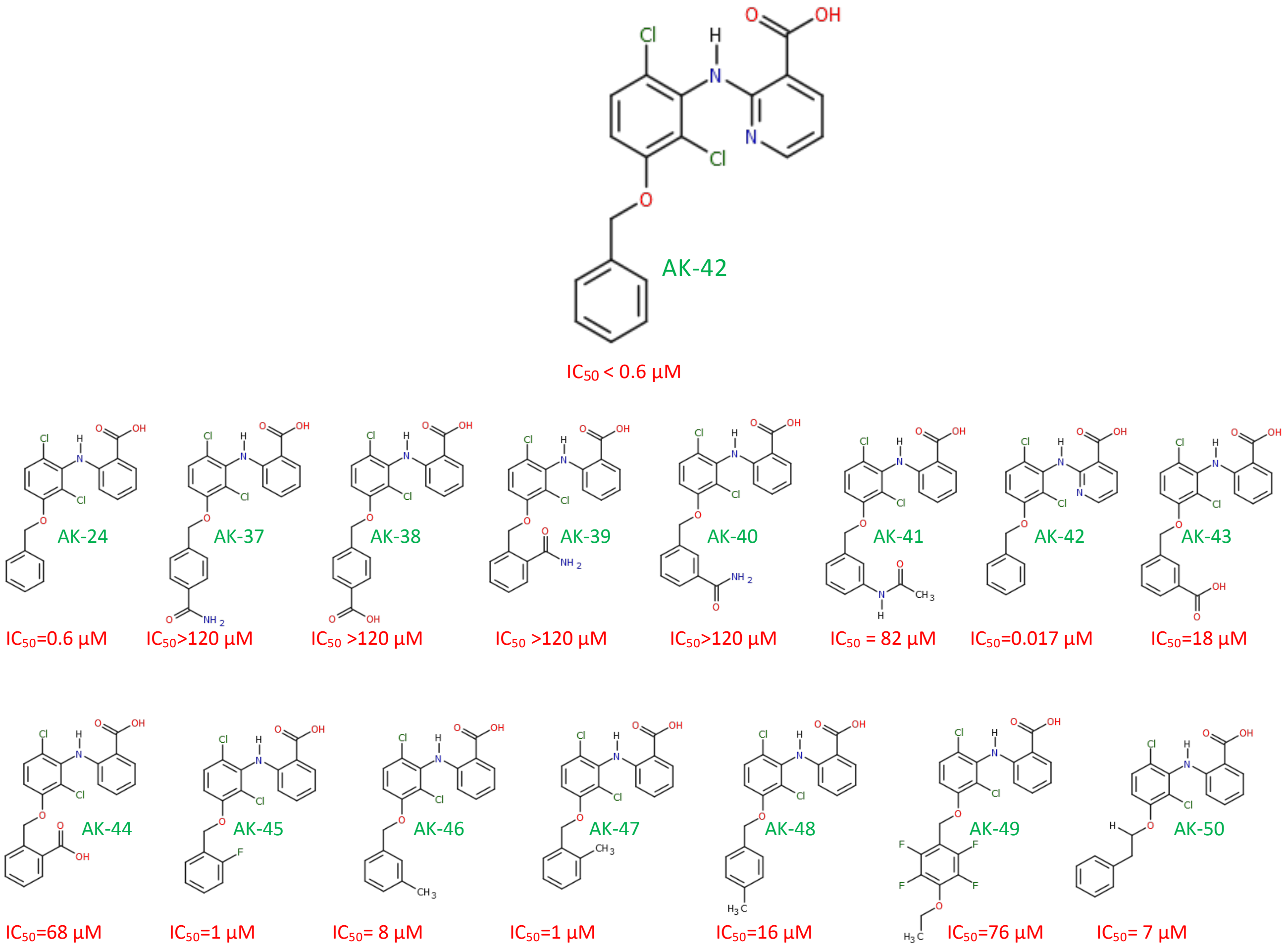

Supplementary Figure 12. Analysis of AK-42 derivatives and their half-maximal inhibitory concentration (IC_{50}) with respect to ClC-2. The main structural differences of AK-42 and its derivatives are highlighted. The IC_{50} data were extracted from the previous report[1] and are shown under the corresponding derivatives.

Schematic diagram of 2-((3-(benzyloxy)-2,6-dichlorophenyl)amino)nicotinic acid synthesis

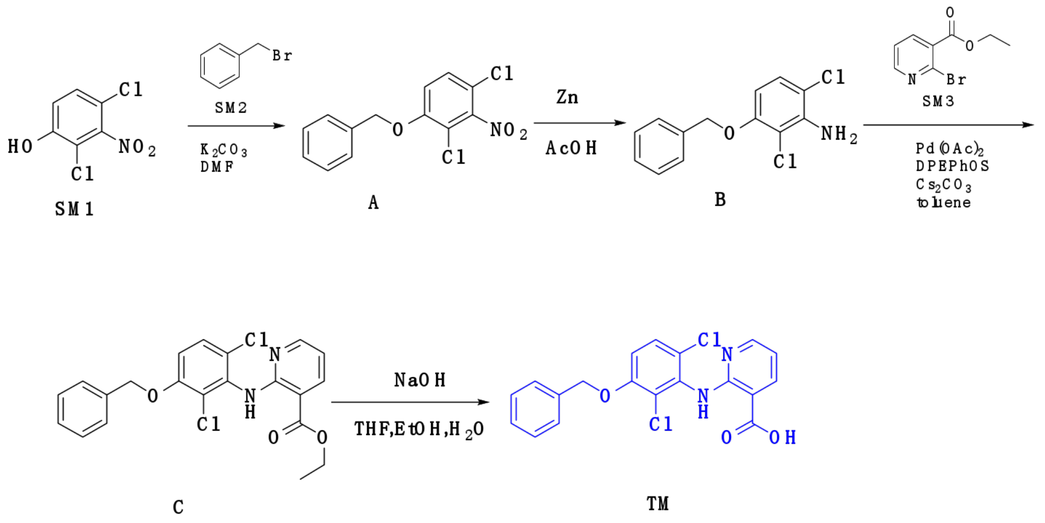

Step 1: 1-(benzyloxy)-2,4-dichloro-3-nitrobenzene

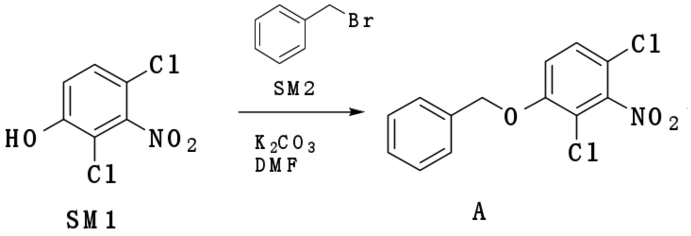

PhBr (SM2, 2.14 g, 12.5 mmol) was added to a mixture of 2,4-dichloro-3-nitrophenol (SM1, 2 g, 9.62 mmol) and K_2CO_3 (2.66 g, 19.24 mmol) in anhydrous DMF (160 mL) in an ice bath. The reaction was stirred overnight at room temperature. After decanting from this ice bath, the product was separated, filtered, and dried to afford the specified product (2.88 g, 98.0%) as a yellow solid. [M+1] = 298

Step 2: 3-(Benzyloxy)-2,6-dichloroaniline

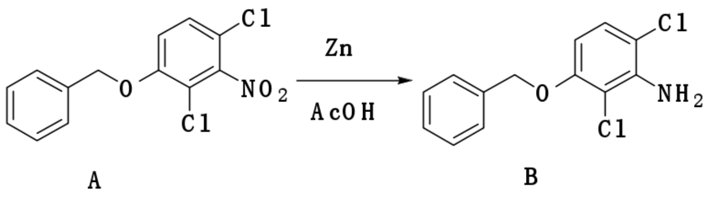

Zn (1.9 g, 29.51 mmol) was added to a mixture of 1-(benzyloxy)-2,4-dichloro-3-nitrobenzene (1.7 g, 5.7 mmol) and AcOH (1.77 g, 29.51 mmol) in anhydrous EtOH (200 mL). The reaction was stirred overnight at room temperature. The organic layer was concentrated, extracted with ethyl acetate and H_2O, dried with anhydrous Na_2SO_4, filtered, and finally concentrated to afford the specified product (1.4 g, 91.5%) as a brown solid. [M+1] = 268.02.

Step 3: <u>methyl 2-((3-(benzyloxy)-2,6-dichlorophenyl)amino)nicotinate</u>

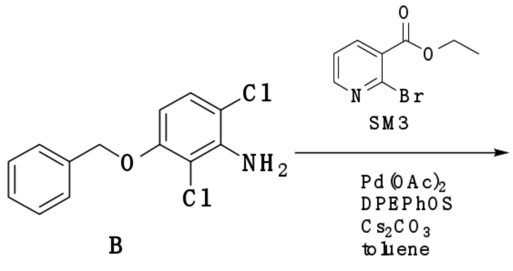

$Pd(OAc)_2$ (33 mg, 0.12 mmol) was added to a mixture of 3-(benzyloxy)-2,6-dichloroaniline (1.0 g, 3.1 mmol), ethyl 2-bromonicotinate (670 mg, 3.1 mmol), DPEPhOS (120 mg, 0.21 mmol) and Cs_2CO_3 (1.3 g, 5.2 mmol) in anhydrous toluene (10 mL). This reaction was stirred for 24 h at 120°C under Ar. The organic layer was filtered, concentrated, and purified by column chromatography with silica gel petroleum ether:ethyl acetate = 8:1 to afford the product (1.2 g, 92.8%) as a white solid. [M+1] = 417.07.

Step 4: <u>2-((3-(benzyloxy)-2,6-dichlorophenyl)amino)nicotinic acid</u>

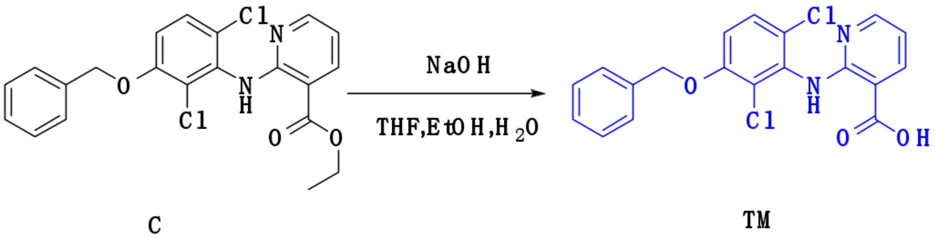

NaOH (336 mg, 8.4 mmol) was added to a mixture of methyl 2-((3-(benzyloxy)-2,6-dichlorophenyl)amino)nicotinate (1.2 g, 2.8 mmol) in THF (5 mL), EtOH (5 mL) and H_2O (1 mL). This reaction was stirred for 2 h at room temperature. The organic layer was concentrated and purified by column

chromatography on silica gel petroleum ether/EA = 3:1 to afford the product (1.02 g, 93.5%) as a white solid. The product was further verified via NMR spectrometry. 1H NMR (400 MHz, DMSO-d6) δ 13.57 (s, 1H), 9.65 (s, 1H), 8.20 (m, 2H), 7.48 (m, 3H), 7.42 (m, 2H), 7.35 (m, 1H), 7.22 (d, J = 9.1 Hz, 1H), 6.83 (m, 1H), 5.25 (s, 2H). [M+1] = 389.04.

Supplementary Figure 13. Schematic of synthesis.

Supplementary Table 1. Summary of Cryo-EM data collection, data processing, and structure refinement. The hardware, software and parameters used are summarized in the table. The validation results are also included.

**Data collection**		
EM equipment	Titan Krios (Thermo Fisher)	
Voltage (kV)	300	
Detector	Gatan K2 Summit	
Energy filter	Gatan GIF, 20 eV slit	
Pixel size (Å)	0.829	
Total electron dose (e^{-} $Å^{-2}$)	50	
Defocus range (μm)	-1.0~-2.0	
**3D Reconstruction**		
Software	Relion-3.0/cryoSPARC	
Data set	ClC-2(apo)	ClC-2(AK-42)
Number of micrographs	7,374	8,951
Final particles	43,510	44,153
Symmetry	C2	C2
Final resolution (Å)	3.5	3.5
Map sharpening B-factor ($Å^2$)	-149	-95
**Refinement**		
Software	Phenix	
**Model composition**		
Protein residues	944	944
Ligand	0	2
R.M.S. deviations		
Bond lengths (Å)	0.002	0.002
Bond angles (°)	0.542	0.579
MolProbity score	1.45	1.59
Clash score	8.32	8.53
Rotamer outliers	0	0
**Ramachandran plot statistics (%)**		
Preferred	98.09	97.34
Allowed	1.91	2.66
Outliers	0	0

Supplementary Table 2. Inhibition kinetics of wild-type ClC-2 and its mutants by AK-42. %baseline represents the currents remained after 3-min application of 30 nM AK-42, with reference to that before application of AK-42. Data are presented as the mean ± SEM. N values represent biologically independent cells recorded. For those responses remained less than 95% of baseline, τ of decay is calculated with single exponential function and represents the time constant of current change during application of AK-42. R-squared R^2 is the goodness-of-fit for the fitting between experimental data and fitting curve as shown in Supplementary Fig. 9.

**ClC-2 plasmids**	**% baseline (30 nM AK-42)**	**τ of decay/R^2**
WT	42.966 ± 5.076 (n = 6)	0.9444/0.8407
D109K	74.148 ± 5.667 (n = 4)	3.693/0.5442
K204M	79.386 ± 3.869 (n = 3)	1.949/0.8002
K204R	69.890 ± 0.528 (n = 3)	1.285/0.9604
F306Y	93.394 ± 5.940 (n = 4)	0.8136/0.1650
S392A	75.704 ± 3.740 (n = 3)	1.222/0.7956
Q393P	80.994 ± 5.580 (n = 3)	1.255/0.6738
K394A	53.917 ± 2.613 (n = 3)	4.771/0.8329
K394R	45.652 ± 5.943 (n = 3)	1.217/0.9414
L397S	53.217 ± 8.881 (n = 3)	0.6740/0.9001
F459A	18.768 ± 2.172 (n = 3)	0.4764/0.9861
M460A	53.768 ± 5.776 (n = 4)	0.9606/0.8809
M460K	79.383 ± 2.164 (n = 4)	1.215/0.8991
M460W	96.945 ± 1.874 (n = 4)	-/-
F463A	60.738 ± 10.137 (n = 4)	0.8627/0.7519
F463L	92.465 ± 4.515 (n = 4)	2.379/0.3685
F463M	84.893 ± 1.484 (n = 3)	1.951/0.8703
WT+DMSO	96.803 ± 2.635 (n = 5)	-/-

Supplementary Table 3. Current inhibition induced by AK-42 (30 nM) treatment in ClC-2 wild-type and mutants. The percentage current inhibition was calculated as follows: percent inhibition = ($I_{initial}$-I_{AK})/$I_{initial}$ X 100%, where $I_{initial}$ and I_{AK} are the currents measured before and after treatment with 30 nM AK-42, respectively, at -100 mV. Data are presented as the mean ± SEM. Exact n values and P values are listed. N values represent biologically independent cells recorded. Statistical analyses of percent inhibition between WT and mutants were performed by two-tailed Student's t test. The data are plotted in Figure 3g.

ClC-2 plasmids	% inhibition (30 nM AK-42)	Student's t-test (with WT)
WT	61.68 ± 3.896 (n = 14)	
D109K	32.09 ± 7.026 (n = 6)	P = 0.0009, t = 3.949
K204M	18.83 ± 5.293 (n = 3)	P = 0.0002, t = 4.818
K204R	20.71 ± 1.668 (n = 6)	P < 0.0001, t = 6.676
F306Y	2.335 ± 0.8473 (n = 3)	P < 0.0001, t = 6.868
S392A	28.22 ± 4.445 (n = 4)	P = 0.0005, t = 4.310
Q393P	32.32 ± 5.164 (n = 7)	P = 0.0003, t = 4.436
K394A	56.19 ± 2.743 (n = 5)	P = 0.4325, t = 0.8040
K394R	48.34 ± 5.534 (n = 10)	P = 0.0543, t = 2.033
L397S	62.41 ± 4.279 (n = 5)	P = 0.9180, t = 0.1045
F459A	75.19 ± 3.453 (n = 6)	P = 0.0497, t = 2.104
M460A	52.30 ± 7.118 (n = 6)	P = 0.2287, t = 1.246
M460K	32.58 ± 8.185 (n = 5)	P = 0.0022, t = 3.595
M460W	17.94 ± 4.528 (n = 5)	P < 0.0001, t = 6.145
F463A	43.16 ± 4.581 (n = 5)	P = 0.0188, t = 2.598
F463L	5.983 ± 1.994 (n = 4)	P < 0.0001, t = 7.412
F463M	20.55 ± 5.162 (n = 6)	P < 0.0001, t = 5.991

Supplementary Table 4. Primers used in this study.

ClC-2 mutants	Forward primers (5' to 3')	Reverse primers (5' to 3')
D109K	TGGGTCATGAAATATGCCATT	AATGGCATATTTCATGACCCA
G203A	ATGCCGCTTGCCAAAGAGGGC	GCCCTCTTTGGCAAGCGGCAT
K204M	CTTGGCATGGAGGGCCCTTTT	AAAAGGGCCCTCCATGCCAAG
K204R	ATGCCGCTTGGCAGAGAGGGC	GCCCTCTCTGCCAAGCGGCAT
F306N	ATTACAGCCCTCAACAAAACCCGA	TCGGGTTTTGTTGAGGGCTGTAAT
F306Y	ATTACAGCCCTCTACAAAACCCGA	TCGGGTTTTGTAGAGGGCTGTAAT
S392A	GCTGGACAGCTCGCACAGAAAGAG	CTCTTTCTGTGCGAGCTGTCCAGC
Q393P	GACAGCTCTCACCAAAAGAGACGCTG	CAGCGTCTCTTTTGGTGAGAGCTGTC
K394A	CAGCTCTCACAGGCAGAGACGCTG	CAGCGTCTCTGCCTGTGAGAGCTG
K394R	CAGCTCTCACAGAGAGAGACGCTG	CAGCGTCTCTCTCTGTGAGAGCTG
L397S	AAAGAGACGTCGGTCACCCTG	CAGGGTGACCGACGTCTCTTT
F459A	TGTGGGGCCGCAATGCCTGTCTTT	AAAGACAGGCATTGCGGCCCCACA
M460A	GCCTTCGCACCTGTCTTTGTCATTG	CAATGACAAAGACAGGTGCGAAGGC
M460K	GCCTTCAAGCCTGTCTTTGTCATTG	CAATGACAAAGACAGGCTTGAAGGC
M460W	GCCTTCTGGCCTGTCTTTGTCATTG	CAATGACAAAGACAGGCCAGAAGGC
F463A	TTCATGCCTGTCGCTGTCATTGGA	TCCAATGACAGCGACAGGCATGAA
F463L	TTCATGCCTGTCCTTGTCATTGGA	TCCAATGACAAGGACAGGCATGAA
F463M	TTCATGCCTGTCATGGTCATTGGA	TCCAATGACCATGACAGGCATGAA
L524S	ATCGTGTTCGAGTCCACAGGCCAG	CTGGCCTGTGGACTCGAACACGAT

Supplementary Table 5. Current inhibition induced by vehicle and AK-42 (30 nM) in wild-type ClC-2. AK-42 was dissolved in and diluted to a final concentration of 30 nM in 0.1% DMSO in extracellular solution as a vehicle. Measurement of the percentage of current inhibition before and after treatment with 0.1% DMSO or 30 nM AK-42 at -100 mV. Data are presented as the mean ± SEM.

ClC-2 WT	percent inhibition
+ vehicle (0.1% DMSO)	2.909 ± 1.441 (n = 4)
+ AK-42 (30 nM)	61.68 ± 3.896 (n = 14)

Supplementary References

1. Koster AK, *et al.* Development and validation of a potent and specific inhibitor for the ClC-2 chloride channel. *Proc Natl Acad Sci U S A* **117**, 32711-32721 (2020).
