# Supplementary material for: Cryo-EM structures of ClC-2 chloride channel reveal the blocking mechanism of its specific inhibitor AK-42
Source: Nat Commun. 2023 Jun 9;14:3424. doi: 10.1038/s41467-023-39218-6 (PMC10256776; doi:10.1038/s41467-023-39218-6)
Supplement: Supplementary file 2 — Reporting Summary [file 41467_2023_39218_MOESM2_ESM.pdf]

nature portfolio

Corresponding author(s): Huawei Zhang

Last updated by author(s): May 13, 2022

Reporting Summary

Nature Portfolio wishes to improve the reproducibility of the work that we publish. This form provides structure for consistency and transparency in reporting. For further information on Nature Portfolio policies, see our Editorial Policies and the Editorial Policy Checklist.

Statistics

For all statistical analyses, confirm that the following items are present in the figure legend, table legend, main text, or Methods section.

n/a	Confirmed	
☐	☒	The exact sample size (*n*) for each experimental group/condition, given as a discrete number and unit of measurement
☐	☒	A statement on whether measurements were taken from distinct samples or whether the same sample was measured repeatedly
☐	☒	The statistical test(s) used AND whether they are one- or two-sided<br>*Only common tests should be described solely by name; describe more complex techniques in the Methods section.*
☒	☐	A description of all covariates tested
☐	☒	A description of any assumptions or corrections, such as tests of normality and adjustment for multiple comparisons
☐	☒	A full description of the statistical parameters including central tendency (e.g. means) or other basic estimates (e.g. regression coefficient) AND variation (e.g. standard deviation) or associated estimates of uncertainty (e.g. confidence intervals)
☐	☒	For null hypothesis testing, the test statistic (e.g. F, t, r) with confidence intervals, effect sizes, degrees of freedom and P value noted<br>*Give P values as exact values whenever suitable.*
☒	☐	For Bayesian analysis, information on the choice of priors and Markov chain Monte Carlo settings
☒	☐	For hierarchical and complex designs, identification of the appropriate level for tests and full reporting of outcomes
☒	☐	Estimates of effect sizes (e.g. Cohen's *d*, Pearson's *r*), indicating how they were calculated

Our web collection on statistics for biologists contains articles on many of the points above.

Software and code

Policy information about availability of computer code

Data collection	Cryo-EM: SerialEM(v3.5). Patch clamp experiment: Clampex v11.2 (Molecular Devices) and Clampfit v11.2 (Molecular Devices).
Data analysis	CryoEM Image Analysis Software: MotionCor2(v1.3.1), CTFFIND(v4.1), Relion(v3.1.2), Cryosparc(v3.3.1); Atomic modeling and visualization: Coot(v0.8.9), Phenix(v1.20.1), Molprobility(v4.5), Chimera(v1.16), PyMOL(v2.5.2), CAVER(v3.0.3); Statistical Analysis: GraphPad Prism(v9.0.0); Molecular dynamics simulations: CHARMMGUI (v3.0), Gromacs (v2019), VMD (v1.9.3), XMGRACE (5.1.25).

For manuscripts utilizing custom algorithms or software that are central to the research but not yet described in published literature, software must be made available to editors and reviewers. We strongly encourage code deposition in a community repository (e.g. GitHub). See the Nature Portfolio guidelines for submitting code & software for further information.

Data

Policy information about availability of data

All manuscripts must include a data availability statement. This statement should provide the following information, where applicable:

- Accession codes, unique identifiers, or web links for publicly available datasets
- A description of any restrictions on data availability
- For clinical datasets or third party data, please ensure that the statement adheres to our policy

The cryo-EM maps of human ClC-2 were deposited in the Electron Microscopy Data Bank under accession numbers EMD-33169 [https://www.ebi.ac.uk/pdbe/entry/emdb/EMD-33169] (Apo full length), EMD-33223 [https://www.ebi.ac.uk/pdbe/entry/emdb/EMD-33223] (Apo TMD domain) and EMD-34202 [https://www.ebi.ac.uk/pdbe/entry/emdb/EMD-34202] (AK-42 bound TMD domain). The corresponding atomic coordinates were deposited in the RCSB Protein Data Bank

under accession numbers 7XF5 [https://doi.org/10.2210/pdb7XF5/pdb], 7XJA [https://doi.org/10.2210/pdb7XJA/pdb] and 8GQU [https://doi.org/10.2210/pdb8GQU/pdb], respectively. Atomic coordinates for human ClC-1, bovine ClC-K and human ClC-7 mentioned in this work can be accessed with PDB codes 6COY [https://doi.org/10.2210/pdb6COY/pdb], 5TQQ [https://doi.org/10.2210/pdb5TQQ/pdb] and 7JM7 [https://doi.org/10.2210/pdb7JM7/pdb] respectively. The source data underlying Figures 2c, 3g and Supplementary Figures 1, 7, 11b are provided as a Source Data file.

Human research participants

Policy information about studies involving human research participants and Sex and Gender in Research.

Reporting on sex and gender	N/A
Population characteristics	N/A
Recruitment	N/A
Ethics oversight	N/A

Note that full information on the approval of the study protocol must also be provided in the manuscript.

Field-specific reporting

Please select the one below that is the best fit for your research. If you are not sure, read the appropriate sections before making your selection.

- [x] Life sciences
- [ ] Behavioural & social sciences
- [ ] Ecological, evolutionary & environmental sciences

For a reference copy of the document with all sections, see nature.com/documents/nr-reporting-summary-flat.pdf

Life sciences study design

All studies must disclose on these points even when the disclosure is negative.

Sample size	CryoEM sample size were not predetermined. An initial dataset of 7,374 micrographs for ClC-2 apo state and 8, 951 micrographs for AK-42 bound state (1,612,725 and 1,741,519 particles respectively) was collected and used to assess the effects of particle number on the achievable resolution and/or resolvability of particle features during 3D classification. The initial size was estimated based on the particle density observed in test images. The particles size of initial dataset was sufficient to achieve fine resolution of the resulting CryoEM maps, indicating the size of the particles were sufficiently sampled.<br>For patch clamp experiments, No statistical method was used to predetermine sample sizes, but sample sizes are similar to previous publication(Koster et al.,PNAS,2020).
Data exclusions	For cryo-EM studies, single particle image data was excluded based on the absence of high-resolution features (e.g. alpha-helical transmembrane domains), which were conditions that had been pre-established based on the structures of homologous proteins.<br>For patch clamp experiments, experiments in which initial currents induced at the -100 mV to be larger than 100 pA and inhibition calculated using initial currents differed from inhibition calculated using washout currents by less than 30% were included for analyses.
Replication	For cryo-EM studies, all attempts at replication were successful. This included processing two independent datasets obtained from unique particles in test dataset and initial dataset, and by processing with alternative CryoEM image analysis software (Relion (v3.1.2)).<br>For patch clamp experiments, two or three batches of cells for each mutation group were tested.
Randomization	For cryo-EM studies, Single particle image data was split randomly into two groups and processed in the same way to calculate Fourier-shell correlation coefficients, in accordance to Gold Standard Methods. Samples were not further allocated into groups, outside of what is performed by the computational image analysis programs used in this work.<br>For patch clamp experiments, cells with GFP fluorescence were randomly chosen for whole-cell recordings.
Blinding	Investigators were not blinded during data acquisition, experiment conduction or analysis. Blinded studies in this case were not possible because the investigator performed the experiments and analysis contributed by the isolation of the specimen or cells.

Reporting for specific materials, systems and methods

We require information from authors about some types of materials, experimental systems and methods used in many studies. Here, indicate whether each material, system or method listed is relevant to your study. If you are not sure if a list item applies to your research, read the appropriate section before selecting a response.

Materials & experimental systems

n/a	Involved in the study
x	Antibodies
	x Eukaryotic cell lines
x	Palaeontology and archaeology
x	Animals and other organisms
x	Clinical data
x	Dual use research of concern

Methods

n/a	Involved in the study
x	ChIP-seq
x	Flow cytometry
x	MRI-based neuroimaging

Eukaryotic cell lines

Policy information about cell lines and Sex and Gender in Research

Cell line source(s)	HEK293F (Thermo Fisher) , CHO-K1 (Thermo Fisher)
Authentication	cell lines were not authenticated.
Mycoplasma contamination	cell lines were not tested for Mycoplasma contamination.
Commonly misidentified lines (See ICLAC register)	No commonly misidentified lines were used in this study.
